# Sialic acid–guided spatiotemporal hydrogel therapy for liver cancer

**DOI:** 10.1016/j.mtbio.2026.102784

**Published:** 2026-01-08

**Authors:** Weiqiang Hao, Hyeon Ji Kim, Jumi Kang, Bongkyun Kang, Seoyeon Park, Yuejin Kim, Eunjeong Kim, Kyueui Lee

**Affiliations:** a Department of Chemistry, Kyungpook National University, Daegu 41566, South Korea; b Department of Biology, College of Natural Sciences, Kyungpook National University, Daegu 41566, Republic of Korea; c School of Life Sciences, BK21 FOUR KNU Creative BioResearch Group, Kyungpook National University, Daegu, 41566, Republic of Korea; d KNU Institute of Basic Sciences and KNU G-LAMP Project Group, Kyungpook National University, Daegu 41566, South Korea; e School of Life Science and Biotechnology, Kyungpook National University, Daegu 41566, South Korea; f Center for Genome Engineering, Institute for Basic Science, Daejeon 34126, Republic of Korea; g Biomedical Research Institute, Kyungpook National University Hospital, Daegu 41940, South Korea

**Keywords:** Hepatocellular carcinoma, Hydrogel, Polyphenolic drugs, Drug delivery system

## Abstract

Efficient delivery of plant-derived polyphenolic drugs to tumor sites in hepatocellular carcinoma (HCC) is challenging due to their rapid metabolism and the limited tumor-targeting capacity of current therapeutic strategies. To overcome these limitations, we developed a pH-responsive hydrogel-based drug delivery system (PA–CB) composed of a chitosan backbone functionalized with boronobenzoic acid (CB) and crosslinked with protocatechualdehyde (PA). Within this scaffold, protocatechuic acid (PCA) was incorporated as a model therapeutic agent to demonstrate the platform's ability to achieve controlled, pH-responsive release and to impart anticancer, anti-inflammatory, and antifibrotic effects through the action of the drug. The hydrogel, stabilized via boronate ester and Schiff-base linkages, maintained integrity under physiological conditions while enabling drug markedly enhanced anticancer efficacy in vitro compared to free PCA, including a near-complete reduction of HepG2 cell viability, migration, and colony formation, along with increased apoptosis. This enhanced antitumor efficacy was due to CB-mediated recognition of sialic acid residues on HCC cells, which facilitated tumor-selective accumulation and sustained drug release. Intraperitoneal administration of the hydrogel in an HCC mouse model significantly reduced tumor burden, hepatic inflammation, and fibrosis, while improving liver function markers. Histological assessments confirmed alleviation of liver injury, and quantitative polymerase chain reaction analyses revealed decreased expression of proinflammatory cytokines. Collectively, these results highlight this hydrogel platform as a robust strategy to stabilize phenolic drugs, achieve tumor-targeted delivery, and enable controlled release. These findings highlight its potential as an advanced therapeutic approach for HCC and a versatile framework applicable to other polyphenolic agents in oncology.

## Graphical abstract

**Figure:**
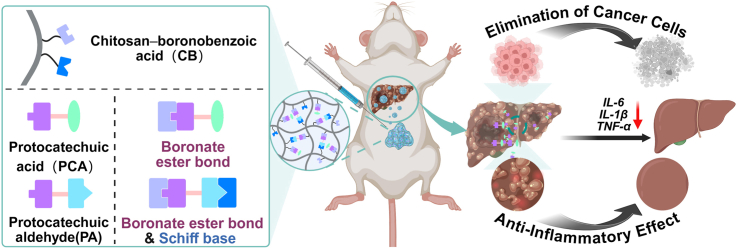

## 1 Introduction

Hepatocellular carcinoma (HCC), the most common form of primary liver cancer, ranks among the leading causes of cancer-related mortality worldwide [1]. Its aggressive biological behavior—marked by rapid progression, early metastatic potential [2], and frequent late-stage diagnosis [3,4]—contribute to its poor prognosis [5,6]. Despite advances in therapeutic strategies—including surgical resection, liver transplantation, locoregional therapies, and systemic treatments [7]—outcomes remain unsatisfactory, especially in advanced stages of the disease. Challenges such as drug resistance [8], treatment-related toxicities, and the limited long-term efficacy [9] underscore the urgent need for safer, more effective therapeutic options. In particular, agents capable of targeting the inflammatory tumor microenvironment—a hallmark of HCC—need to be developed.

Currently, systemic therapy for HCC mainly relies on multitarget kinase inhibitors (e.g., sorafenib and lenvatinib) and immune checkpoint inhibitors (e.g., pembrolizumab and nivolumab) [10,11]. Although sorafenib and lenvatinib have been widely used as first-line agents in the treatment of HCC and can moderately prolong survival, their efficacy remains limited and is often accompanied by severe side effects, including hand–foot skin reactions, diarrhea, fatigue, and impaired liver function [10,12]. Moreover, to the rapid development of drug resistance in HCC [13] further compromises the sustainability of these therapies. On the other hand, immune therapy drugs, especially pembrolizumab, have demonstrated some promise, yet their use is constrained by serious immune-related adverse reactions [14]. Collectively, these limitations highlight an urgent need to develop alternative therapeutic drugs with improved efficacy and fewer side effects.

Plant-derived polyphenols have gained considerable attention as potential new therapeutic agents for HCC, given their intrinsic anti-inflammatory, anticancer, and antioxidant properties, coupled with low toxicity [15,16]. Since chronic inflammation is a prominent feature of the HCC tumor microenvironment, these compounds possess the potential to markedly improve treatment outcomes and emerge as alternatives to conventional therapies [17,18]. Mechanistically, polyphenols exert anticancer effects through multiple pathways; for instance, quercetin [19] and apigenin [20] inhibit tumor-promoting signaling pathways, while protocatechuic acid (PCA) induces apoptosis [21]. However, due to their short half-lives in vivo (quercetin = ∼3.5 h [22], apigenin = ∼2.5 h [23], and PCA = ∼16 min [24]), these compounds are prone to rapid metabolism and clearance. Moreover, delivery strategies based on simple physical entrapment provide limited control over small-molecule polyphenols, which can cause premature leakage and reduced exposure at the disease site. Many hydrogel carriers also lack active recognition elements and thus rely largely on passive accumulation, further limiting tumor-selective delivery. These limitations underscore the urgent need for innovative drug delivery systems (DDS) designed to enhance the bioavailability, stability, and tumor-targeting capability of plant-derived polyphenols, thereby advancing their utility in effective HCC treatment.

In this study, we developed a pH-responsive hydrogel system for the sustained and controlled release of plant-derived polyphenols. Chitosan was functionalized with 3-boronobenzoic acid (BA) to form a boronic-acid-modified scaffold (CB), onto which PCA, the model drug used in this study, was loaded through reversible interactions between its catechol groups and the scaffold's boronic acid moieties. To further enhance structural stability and environmental responsiveness, protocatechualdehyde (PA), a dual-functional crosslinker capable of forming reversible boronate ester bonds via its catechol group and dynamic Schiff-base linkages via its aldehyde group, was incorporated into the scaffold. This dual crosslinking endowed the hydrogel with structural integrity while enabling pH-responsive release of PCA in the acidic tumor microenvironment of HCC (pH ≈ 6.5). In vivo studies demonstrated that intraperitoneal administration of the PA–CB–PCA system significantly enhanced drug retention at tumor sites, inhibited HCC tumor growth, and modulated gene expression—downregulating proinflammatory cytokines and oncogenes while upregulating tumor suppressor genes—thereby exerting antitumor effects through multiple synergistic mechanisms. In summary, the PA–CB–PCA hydrogel is a structurally robust, highly responsive, and broadly adaptable platform that allows tumor-targeted delivery of phenolic drugs, while enhancing the therapeutic efficacy and clinical translatability of these agents.

## 2 Experimental section

### 2.1 Materials

1-(3-Dimethylaminopropyl)-3-ethylcarbodiimide hydrochloride (EDC·HCl), protocatechuic acid (PCA), and N-acetylneuraminic acid (Neu5Ac, the most common form of sialic acid, hereafter referred to as SA) were purchased from Tokyo Chemical Industry Co., Ltd. (Tokyo, Japan). N-hydroxysuccinimide (NHS), 3-boronobenzoic acid (BA), chitosan (CS; low molecular weight), dimethylformamide (DMF; ≥99.8 %), sodium hydroxide (NaOH), protocatechualdehyde (PA), deuterium oxide (D_2_O), dimethyl sulfoxide-d6 (DMSO-d6), fluorescein isothiocyanate (FITC) isomer I, and crystal violet were obtained from Sigma-Aldrich (St. Louis, MO, USA). Hydrochloric acid (HCl; 1N) was obtained from Yakuri Pure Chemicals Co., Ltd. (Kyoto, Japan). 2,2-Diphenyl-1-picrylhydrazyl (DPPH), methanol, and the LIVE/DEAD™ Viability/Cytotoxicity Kit were purchased from Thermo Fisher Scientific (Waltham, MA, USA). Sodium chloride (NaCl) was obtained from Samchun Chemicals (Pyeongtaek, South Korea).

The HepG2 (human hepatocellular carcinoma) and L929 (murine fibroblast) cell lines were obtained from the American Type Culture Collection (ATCC, Manassas, VA, USA). Dulbecco's modified Eagle medium (DMEM, high glucose), fetal bovine serum (FBS), penicillin–streptomycin, 0.25 % trypsin solution, and Dulbecco's phosphate-buffered saline (DPBS) were purchased from Cytiva (Marlborough, MA, USA). The Cell Counting Kit-8 (CCK-8) was obtained from Dojindo Molecular Technologies, Inc. (Kumamoto, Japan).

### 2.2 Synthesis of CB

The CB scaffold was synthesized via amide bond formation between the amino groups of CS and the carboxyl groups of BA, using EDC·HCl and NHS as coupling agents. The molar amount of CS was calculated on the basis of glucosamine repeat units and primary amines (–NH_2_) (80 % deacetylation and an average residue molecular weight of ∼169.4 g/mol). In brief, 500 mg of CS (containing approx. 2.36 mmol of free amino groups) was dissolved in 40 mL of 0.05 M HCl solution. Separately, 0.181 g (1.09 mmol) of BA was dissolved in 4 mL of DMF, 0.21 g (1.10 mmol) of EDC·HCl was dissolved in 1 mL of deionized water (DW), and these solutions were mixed and stirred for 10 min to initiate the carboxyl activation, followed by the addition of 0.13 g (1.13 mmol) of NHS (dissolved in 1 mL of DMF) and continuous stirring for another 1 h to form the stable NHS-activated esters. The corresponding molar feed ratio of –COOH (BA): EDC: NHS: NH_2_ (CS) was approximately 1 : 1.01: 1.04 : 2.17. The activated BA solution was then slowly added dropwise to the CS solution, and the pH was adjusted to 6 using 0.5 M NaOH. The mixture was stirred at room temperature for 21 h. After reaction completion, the mixture was centrifuged to remove precipitates and the supernatant was dialyzed against 4 g/L NaCl solution using Spectra/Por® 6 standard regenerated cellulose dialysis tubing, MWCO 6–8 kDa (Repligen Corporation, Waltham, MA, USA), with the dialysis solution replaced every 18 h for 3 days. On the final day, DW was used for further purification. The dialyzed product was frozen and lyophilized for 3 days to yield the white CB product.

### 2.3 Formation of the PA–CB–PCA hydrogel

To prepare the PA–CB–PCA drug–hydrogel composite, CB (10 mg) was dissolved in 200 μL of PBS (pH 7.4). Based on the degree of substitution of BA on CB (DOS = 10.8 %), 10 mg of CB corresponds to 5.83 μmol of boronic acid groups. Subsequently, PCA solution (100 μL, 19.5 mg/mL; 12.65 μmol) and PA solution (50 μL, 1 mg/mL; 0.362 μmol) were sequentially added, and the mixture was stirred at room temperature for 5 min to form the hydrogel. The feed molar ratio of boronic acid groups:PCA:PA was 1.00:2.17:0.06 (total mixing volume, 350 μL). Because PCA and PA each provide one catechol moiety, the above molar amounts correspond to catechol equivalents. PCA was added in molar excess to drive reversible boronate ester formation forward and achieve the targeted loading, while any unreacted fraction may remain physically entrapped within the network.

### 2.4 Characterization of PA–CB–PCA hydrogel

^1^H NMR spectra were recorded on a Bruker AVANCE III 500 spectrometer (Bruker BioSpin GmbH, Rheinstetten, Germany). CB, PCA, SA, CB–PCA, and CB–SA were each dissolved in 1 mL of D_2_O for testing; CS was dissolved in D2O with 1 drop of acetic acid, and BA was dissolved in DMSO-d6.

FT-IR spectra were obtained using a Perkin Elmer Frontier spectrometer (Perkin Elmer, Shelton, CT, USA) over a 4000–400 cm^−1^ range. CB and CB–SA were analyzed in the form of compressed films, while CB–PCA and PA–CB–PCA were tested as lyophilized powders. CS, SA, and BA were directly analyzed in their powder form.

### 2.5 Rheological studies

Rheological measurements were performed using an MCR-92 modular compact rheometer (Anton Paar, Graz, Austria) equipped with a parallel-plate geometry (diameter 25 mm) to record storage modulus (G′) and loss modulus (G″) at 23 °C and 37 °C. Viscosity tests were conducted at shear rates of 1–10 s^−1^. Strain sweeps were performed at a fixed frequency of 5 rad/s with strains from 0.1 % to 100 %. Frequency sweeps were carried out at a fixed oscillatory strain of 1 % over frequencies from 0.1 to 100 rad/s.

### 2.6 Cytotoxicity assay

The cytotoxicity of the PA–CB–PCA hydrogel against HepG2 cells was evaluated using the CCK-8 assay. HepG2 cells were seeded in 96-well plates (Corning Life Sciences, Tewksbury, MA, USA) at a density of 1 × 10^4^ cells/100 μL per well and cultured in DMEM supplemented with 10 % FBS and 1 % penicillin–streptomycin at 37 °C in a 5 % CO_2_ atmosphere. After 24 h, the cells were treated with PA–CB–PCA extracts (prepared by extraction at 0.5, 1, and 2 mg/mL in DMEM at 37 °C for 24 h, followed by filtration). The medium was refreshed at 48 h. After a 72-h incubation period, 10 μL of CCK-8 solution was added per well, and cells were incubated for an additional 3 h. Absorbance was measured at 450 nm using an Infinite® 200 Pro microplate reader (Tecan, Männedorf, Switzerland), and cell viability was calculated.

### 2.7 In vitro scratch assay

HepG2 cell migration in response to the PA–CB–PCA hydrogel was assessed using a scratch assay. The cells (5 × 10^5^ cells/well) were seeded in 6-well plates and cultured to 80–90 % confluence at 37 °C in 5 % CO_2_. A straight scratch was made using a 200 μL pipette tip, and cells were washed three times with DMEM to remove debris. This was followed by incubation with the PA–CB–PCA extract (1 mg/mL) for 24 h. Migration at 0 and 24 h was imaged using an inverted fluorescence microscope (Nikon TS2-LS, Tokyo, Japan) and compared to controls. Migration area over 24 h in the wound-healing assay was quantified in FIJI and normalized to the control (control = 100 %).

### 2.8 Colony formation assay

The impact of PA–CB–PCA on the proliferative capacity of HepG2 cells was evaluated using a colony formation assay. Cells were exposed to the PA–CB–PCA extract (1 mg/mL) for 24 h and then detached, counted (500 cells), and reseeded in 6-well plates. After 14 days of incubation, colonies were fixed with ice-cold 70 % methanol for 15 min, dried, and stained with 0.5 % crystal violet (diluted in methanol) for 30 min. Subsequently, they were washed with water, dried, photographed using a smartphone, and manually identified and counted.

### 2.9 LIVE/DEAD staining

HepG2 cells (5 × 10^4^ cells/well) were seeded in 24-well plates, stabilized for 24 h at 37 °C in 5 % CO_2_, and then co-cultured with the PA–CB–PCA extract (1 mg/mL). Live/dead staining with calcein-AM/PI was performed on days 0, 1, and 2 using the LIVE/DEAD™ Viability/Cytotoxicity Kit. The stained cells were imaged using a Nikon Eclipse Ts2-LS inverted fluorescence microscope (Nikon, Tokyo, Japan).

### 2.10 In vitro drug-release studies

PCA release from the PA–CB–PCA hydrogels was evaluated using a dialysis method (5.85 mg PCA per hydrogel, n = 3). Each hydrogel was placed in a dialysis membrane (MWCO 6–8 kDa) containing 10 mL of PBS and immersed in 30 mL of PBS (pH 7.4 or 6.5) in a shaking incubator (100 rpm) at 37 °C. At predetermined time points, 1 mL of the external medium was withdrawn and immediately replaced with an equal volume of fresh PBS. PCA quantification was performed by HPLC using a Shimadzu Nexera system and an Agilent ZORBAX Phenyl column (phenyl-bonded silica, 250 × 4.6 mm, 5 μm) maintained at 40 °C. The mobile phase consisted of water (A) and acetonitrile (B), each containing 0.01 % (v/v) TFA, delivered isocratically at 70:30 (A:B, v/v) at a flow rate of 1.0 mL/min for 45 min. The injection volume was 10 μL, and UV detection was performed at 254 nm with additional monitoring at 280 nm. PCA was quantified using an external calibration curve.

### 2.11 Drug release kinetics analysis

The PCA release kinetics from PA–CB–PCA were analyzed using the zero-order, first-order, Higuchi, and Korsmeyer–Peppas models, with curve fitting performed using OriginPro 2021 (64-bit, v9.8.0.200; SR0; OriginLab Corporation, Northampton, MA, USA). In these models, $M_{t}$, $M_{\infty }$, $M_{t}/M_{\infty }$, k, and n denote the cumulative amount released at time t, the cumulative release at infinite time, the fractional release at time t, the kinetic constant, and the release exponent, respectively. The release mechanism was determined from the n value of the Korsmeyer–Peppas model: n < 0.45 indicates Fickian diffusion, 0.45 < n < 0.89 indicates a combination of diffusion and polymer relaxation, and n > 0.89 indicates super case II transport.

(1) $$\text{Zero}-\text{order}:\frac{M_{t}}{M_{\infty }}=\text{kt}$$

(2) $$\text{First}‐\text{order}:\ln\left(1‐\frac{M_{t}}{M_{\infty }}\right)=‐\text{kt}$$

(3) $$\text{Higuchi}:\frac{M_{t}}{M_{\infty }}=kt^{1/2}$$

(4) $$\text{Korsmeyer}–\text{Peppas}:\frac{M_{t}}{M_{\infty }}=kt^{n}$$

### 2.12 DPPH radical-scavenging assay for antioxidant activity of the PA–CB–PCA hydrogel

The antioxidant activity of the PA–CB–PCA hydrogel was evaluated using the DPPH radical-scavenging assay at 37 °C. Lyophilized hydrogel samples (0.5, 1, 3, and 5 mg) were accurately weighed and each dispersed in 1.0 mL of freshly prepared DPPH solution (100 μM in ethanol). The mixtures were stirred gently and incubated in the dark at 37 °C for 30 min, after which the absorbance was recorded at 517 nm using a microplate reader (Infinite® 200 Pro, Tecan, Männedorf, Switzerland).

The radical-scavenging rate (Rs) was calculated using the following equation:

(5) $$R_{s}=\frac{A_{b}‐A_{h}}{A_{b}}\times 100\%$$

where Ab is the absorbance of the blank (DPPH + ethanol) and Ah is the absorbance of the hydrogel-containing sample (DPPH + ethanol + PA–CB–PCA).

### 2.13 CB-FITC: synthesis and cancer-cell-specific uptake

CB-FITC was synthesized as follows: 100 mg of CB was dissolved in 10 mL of DW, and 2 mg of FITC was dissolved in 2 mL of ethanol. The two solutions were mixed, stirred overnight at room temperature, dialyzed for 72 h, and lyophilized to obtain CB-FITC. The entire process was conducted under light-protected conditions. A 0.25 mg/mL CB-FITC solution was freshly prepared in DW just before use.

L929 and HepG2 cells were seeded in 24-well plates (5 × 10^4^ cells per well in 0.8 mL of DMEM) and cultured for 24 h. Subsequently, 0.2 mL of freshly prepared CB-FITC solution was added to each well. Cell-free 24-well plates subjected to identical conditions served as controls to account for potential adsorption of CB-FITC by the plate. CB-FITC attachment to the cells was assessed at 1 h, 3 h, and 5 h. After PBS washing and the subsequent addition of 200 μL PBS to maintain moisture, FITC fluorescence images were captured using an inverted fluorescence microscope (Nikon TS2-LS, Nikon, Tokyo, Japan). Fluorescence area and average intensity on the cell surface were quantified using Fiji ImageJ software.

### 2.14 Covalent bonding of CB and SA under tumor microenvironment pH conditions

As the tumor microenvironment typically exhibits a pH of 6.5–6.8, reactions between CB and SA were carried out at pH 6.5 and pH 7.0; N-acetylneuraminic acid was selected as a representative SA compound. In brief, 100 mg of CB was dissolved in 2 mL PBS and 121.1 mg of SA was dissolved in 1 mL of PBS buffer at the respective pH. The corresponding CB and SA solutions were mixed, stirred for 24 h at room temperature, dialyzed at the respective pH against DW for 3 days, and finally lyophilized for 3 days. The resulting products were characterized by ^1^H NMR and FT-IR to confirm the CB–SA binding under the two pH conditions.

### 2.15 Experimental animals

All experiments were performed using 6–8-week-old male C57BL/6J mice (Hyochang Science, Korea) maintained under conventional housing conditions with ad libitum access to food and water. All procedures were approved by the Institutional Animal Care and Use Committee (IACUC) at the Model Animal Research Center, Kyungpook National University (KNU 2024-0109 and KNU 2025-0170).

### 2.16 Evaluation of tumor targeting ability of the hydrogel in HCC-induced mice

HCC was induced in mice via hydrodynamic tail vein injection (HTVI), following the procedure described in a previous study [25]. A 2 mL PBS–plasmid solution was injected via the lateral tail vein of mice within 5–7 s. The injection volume was equivalent to 8–10 % of the body weight. The plasmids included the oncogene NRAS^G12V^ and constructs for the tumor suppressor genes Pten and Tp53, all purified using a PureLink™ Expi Endotoxin-Free Giga Plasmid Purification Kit (A31233, InvitrogenTM, Carlsbad, CA, USA). Following injection, the mice were monitored for signs of stress and allowed to recover on a heating pad.

To evaluate in vivo targeting capability, two types of FITC-labeled CB hydrogels, PA–CB–PCA (with the crosslinker) and CB–PCA (without the crosslinker), were prepared as previously described. Mice were randomly divided into four groups (n = 3 per group): healthy mice injected with PA–CB–PCA, healthy mice injected with CB–PCA, HCC-bearing mice injected with PA–CB–PCA, and HCC-bearing mice injected with CB–PCA. Seven days after tumor induction, mice received an intraperitoneal (i.p.) injection of 350 μL of the indicated hydrogel formulation. Although i.p. injection is not a standard clinical route for HCC, it was used here as a remote administration setting to stringently test whether tumor accumulation arises from BA–SA recognition and whether drug release is triggered by tumor-associated acidity, rather than from forced local placement. The peritoneal cavity provides a large, well-defined space in which the formulation can reproducibly form an in situ depot, reducing procedure-dependent variability during repeated dosing.

Three days after injection, the mice were euthanized and major organs including the liver and kidneys were excised. Fluorescence distribution and intensity were observed and quantified using an IVIS Lumina III in vivo imaging system (Perkin Elmer, USA). Comparisons were made between groups to assess tumor-targeted accumulation and metabolic differences.

### 2.17 Animal experiments and tissue processing

HCC induction in mice followed the procedure outlined in the previous subsection. 7 days after tumor induction, the mice were randomly divided into three groups (n = 4–7/group) and administered intraperitoneal (i.p.) injections three times per week for three consecutive weeks. Group 1 (control) received only PBS, group 2 was administered PA–PCA at a dose of 20 mg/kg, and group 3 received PA–CB–PCA at a dose of 20 mg/kg. Each formulation was dissolved in PBS.

The mice were sacrificed at designated time points, and liver tissues were harvested for subsequent analyses. For RNA extraction, a portion of the liver was snap-frozen in liquid nitrogen and stored at −80 °C. For histological analysis, liver samples were fixed overnight in 4 % paraformaldehyde (PFA) at room temperature.

### 2.18 Serum biochemical assays

Mice were anesthetized with 0.5–2 % isoflurane mixed with oxygen using a tabletop anesthesia machine for approximately 5 min. Adequate anesthesia was confirmed by the absence of a response to painful stimuli. Mice were then euthanized by cervical dislocation, and whole blood was immediately collected via cardiac puncture. The samples were allowed to clot at room temperature for 30 min and centrifuged at 6,000 rpm for 10 min at 4 °C to isolate the serum.

ALT levels were measured using the EnzyChromTM Alanine Transaminase Assay Kit, EALT-100 (BioAssay Systems, Hayward, CA, USA), and total and direct bilirubin levels were measured using the QuantiChromTM Bilirubin Assay Kit (DIBR-180, BioAssay Systems), following the manufacturer's instructions. Absorbance was measured at the recommended wavelengths using a Synergy HTX multimode reader (RRID: SCR_019749; BioTek Instruments, Winooski, VT, USA).

### 2.19 Histological analysis

Fixed liver tissue samples were embedded in paraffin blocks and sectioned (4–5 μm thick). The general tissue morphology and the presence of tumor lesions were assessed using H&E staining. H&E, Sirius Red, and Masson's trichrome staining were performed as described in a previous study [26]. Stained slides were scanned using an Axio Imager A1 microscope (Carl Zeiss Microscopy GmbH, Jena, Germany), and fibrosis was quantified using ImageJ software. Images were converted to 8-bit format, and color thresholding was applied to isolate the stained fibrotic areas (red for Sirius Redand and green for Masson's trichrome). Fibrosis was expressed as the percentage of the positively stained area relative to total tissue area. Data were plotted using GraphPad Prism 9.5.1 (GraphPad Software, Boston, MA, USA). All histological staining was performed by Labcore Co., Ltd. (Seoul, Republic of Korea).

### 2.20 Quantitative real-time polymerase chain reaction (qRT-PCR)

Total RNA was extracted from liver tissue using the RiboExTM (cat. no. 301-001; GeneAll® Biotechnology Co., Ltd., Seoul, Republic of Korea) according to the manufacturer's instructions. cDNA was synthesized from 5 μg of total RNA using the GoScriptTM Reverse Transcriptase kit (A5003; Promega Corporation, Madison, WI, USA). qRT-PCR was performed using SYBR® Green Realtime PCR Master Mix (TOYOBO Co., Ltd.) on a QuantStudioTM 3 Real-Time PCR System (RRID: SCR_020238, Thermo Fisher Scientific). Gene expression levels were normalized to 18S rRNA and calculated using the 2^-ΔΔCt method. Primer sequences are listed in Table 1. All primers were synthesized by Cosmogenetech, Inc. (Seoul, Republic of Korea).

**Table 1:** Primer sequences for qRT-PCR.

Oligonucleotides	Sequence
18S rRNA forward primer	5′-AGTCCCTGCCCTTTGTACACA-3′
18S rRNA reverse primer	5′-CGATCCGAGGGCCTCACTA-3′
TNF-α forward primer	5′-GGTGCCTATGTCTCAGCCTCTT-3′
TNF-α reverse primer	5′-GCCATAGAACTGATGAGAGGGAG-3′
IL-6 forward primer	5′-TACCACTTCACAAGTCGGAGGC-3′
IL-6 reverse primer	5′-CTGCAAGTGCATCATCGTTGTTC-3′
IL-1β forward primer	5′-TGGACCTTCCAGGATGAGGACA-3′
IL-1β reverse primer	5′-GTTCATCTCGGAGCCTGTAGTG-3′

### 2.21 Statistical analysis

Statistical analysis. For datasets with a single control and multiple treatments, we used one-way ANOVA followed by Dunnett's multiple-comparisons test (each treatment vs the control). For the animal experiments, where all pairwise group differences were of interest, we used one-way ANOVA followed by Tukey's multiple-comparisons test. P values are reported as ∗P < 0.05, ∗∗P < 0.01, ∗∗∗P < 0.001, ∗∗∗∗P < 0.0001; ns denotes not significant. All analyses were performed in GraphPad Prism version 9.5.1 (GraphPad Software, Boston, MA, USA).

## 3 Results and discussion

### 3.1 Design rationale and construction of the PA–CB–PCA hydrogel system

**Fig. 1:**
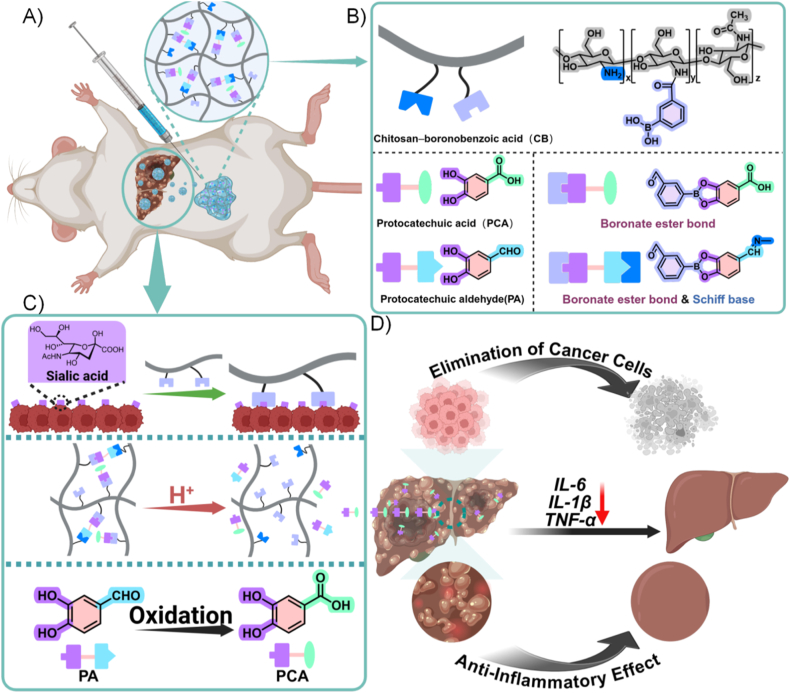
Design of the PA–CB–PCA hydrogel system and its mechanism of action in targeted liver cancer therapy. (A) Schematic illustration of intraperitoneal injection and tumor-targeted accumulation of the hydrogel. (B) Crosslinking and drug-loading through formation of boronate ester and Schiff base linkages between CB, PCA, and PA. (C) In vivo tumor targeting through sialic acid recognition and pH-responsive PCA release; oxidative conversion of PA to PCA. (D) Antitumor effects of the hydrogel system, namely, elimination of cancer cells, downregulation of inflammatory cytokines (IL-6, IL-1β, and TNF-α), and anti-inflammatory effects.

As shown in Fig. 1A, this study developed a hydrogel-based DDS for targeted HCC therapy. CB served as the structural backbone of the hydrogel while also providing active tumor-targeting capability. To reduce the initial burst release commonly observed with physical entrapment, PCA was loaded by forming reversible boronate ester bonds with the boronic-acid-modified chitosan scaffold (Fig. 1B), thereby improving cargo stability prior to reaching the tumor site. PA was incorporated as a dual-functional crosslinker to construct a dual dynamic covalent network through Schiff-base linkages (via its aldehyde group) [27] and boronate ester interactions (via its catechol group), supporting gel integrity under physiological conditions while retaining shear-thinning behavior for injectability. In addition, PA has been reported to undergo oxidative conversion to PCA in vivo (supported by liver-slice and pharmacokinetic studies [[28], [29], [30]]), enabling it to function as a secondary drug source in our system. Finally, CB leverages the affinity between boronic acid groups and sialic acid (SA) residues, enabling active association with HCC cells even under mildly acidic tumor-microenvironment conditions [31]. Collectively, this design provides a structurally robust hydrogel that remains stable under physiological conditions while enabling pH-responsive release in mildly acidic environments.

Given the impracticality of direct intrahepatic injection, intraperitoneal administration was chosen to exploit the tumor-targeting ability of the DDS and achieve selective accumulation in liver tumors. Targeting was mediated by the interaction between boronic acid groups in CB and SA residues highly expressed on the surface of liver cancer cells (Fig. 1C). Once localized at the tumor site, the slightly acidic microenvironment promoted the gradual dissociation of boronate ester bonds between CB and PCA, thereby triggering controlled PCA release and inhibiting tumor cell proliferation. While i.p. injection is not a typical clinical route for HCC, it serves as a reproducible preclinical testbed for mechanism validation in vivo. Direct intrahepatic or peritumoral injection in mice can artificially elevate local exposure and introduce procedure-related artifacts (e.g., tissue injury or leakage) [32], complicating interpretation of active targeting. In addition, absorption from the peritoneal cavity into systemic circulation is generally slower than bolus i.v. dosing [33], supporting repeated dosing while mitigating peak-exposure–driven confounders. Clinically, the BA–SA recognition and acidity-triggered release chemistry tested here could be implemented via liver-directed locoregional routes (e.g., catheter-based intra-arterial delivery/TACE-like approaches or image-guided intrahepatic/peritumoral administration [34,35]), whereas systemic i.v. translation would require a distinct optimization and intravascular safety evaluation pathway.

Consistent with this mechanism, experimental results demonstrated that the drug-loaded hydrogel system significantly enhanced drug retention at the tumor site and effectively reduced both tumor volume and number. Biochemical analysis confirmed that treatment with PA–CB–PCA significantly reduced the expression of proinflammatory cytokines (interleukin [IL]-6, IL-1β, and tumor necrosis factor-alpha [TNF-α]) in the tumor microenvironment, highlighting the phenolic drug's anti-inflammatory effect in vivo (Fig. 1D). As chronic inflammation is known to play a pivotal role in promoting tumor growth and immune evasion, the suppression of these cytokines following treatment is likely a key contributor to the observed anticancer activity [36,37]. In addition, the PA–CB–PCA system was found to upregulate tumor suppressor genes and downregulate oncogene expression, which further highlights the therapeutic potential of this drug–hydrogel formulation. Collectively, these findings suggest that the PA–CB–PCA complex represents a promising strategy for liver cancer therapy, efficiently achieving targeted drug delivery and modulation of tumor-related signaling pathways.

### 3.2 One-step synthesis of CB and crosslinking with PA to form the hydrogel network

**Fig. 2:**
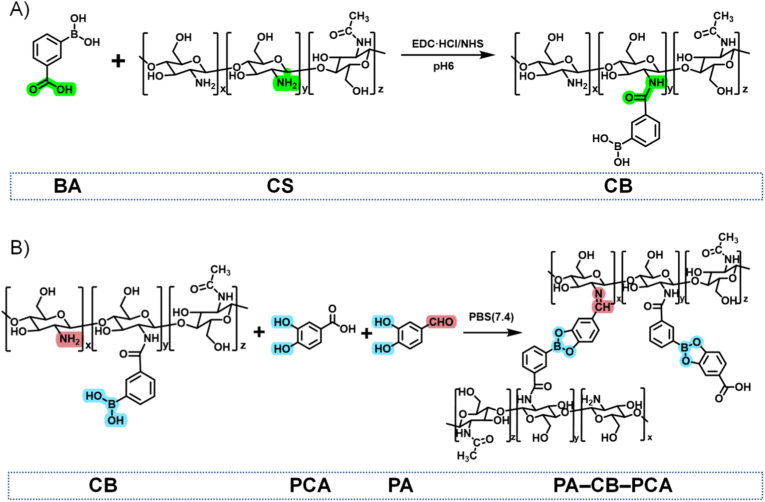
Synthesis and formation of PA–CB–PCA. (A) Synthesis of CB via EDC·HCl/NHS-mediated conjugation of BA onto CS at pH 6. (B) Formation of the drug-loaded hydrogel by mixing CB, PA, and PCA in PBS. CB and PA crosslinked via boronate ester and Schiff-base linkages, while PCA was incorporated into the network through reversible boronate ester interactions.

The detailed synthetic procedure for the CB scaffold is shown in Fig. 2A. CB was easily prepared through a one-step reaction involving EDC/NHS-mediated amide bond formation between the carboxyl groups of BA and the amino groups of CS. The successful incorporation of BA into CS was confirmed using ^1^H nuclear magnetic resonance (NMR) and Fourier transform infrared (FT-IR) spectroscopy (Figs. S1 and S2), and the degree of substitution (DOS) was determined to be 10.8 % (Fig. S3). The improved solubility of the composite material in PBS, a characteristic sign of the chemical modification of CS, further supported the successful grafting of BA onto the CS backbone.

Following the synthesis of CB, PCA and the catechol- and aldehyde-containing crosslinker PA were concurrently incorporated into its framework (Fig. 2B). The boronic acid moieties in the CB scaffold bind PCA via reversible boronate ester bonds, while the primary amines of CB react with the aldehyde groups of PA residues via Schiff-base reactions to yield a robust three-dimensional, dual-crosslinked hydrogel network with enhanced mechanical integrity. The incorporation of PCA into the CB framework was confirmed using ^1^H NMR and FT-IR analyses, with spectral profiles exhibiting peaks corresponding to boronate ester bonds (Figs. S4 and S5). Furthermore, the formation of PA–CB–PCA and the coexistence of both crosslinking modes was confirmed by FT-IR spectra, which exhibited characteristic peaks corresponding to Schiff-base bonds (C=N, 1671 cm^−1^) and B–O–C linkages (1452 cm^−1^) (Fig. S5). Notably, both boronate ester and Schiff-base linkages are pH-responsive, enabling hydrogel degradation and the controlled release of both PCA and PA in the acidic tumor microenvironment, thereby enhancing therapeutic efficacy.

### 3.3 Rheology predicts in vivo stability and injectability

**Fig. 3:**
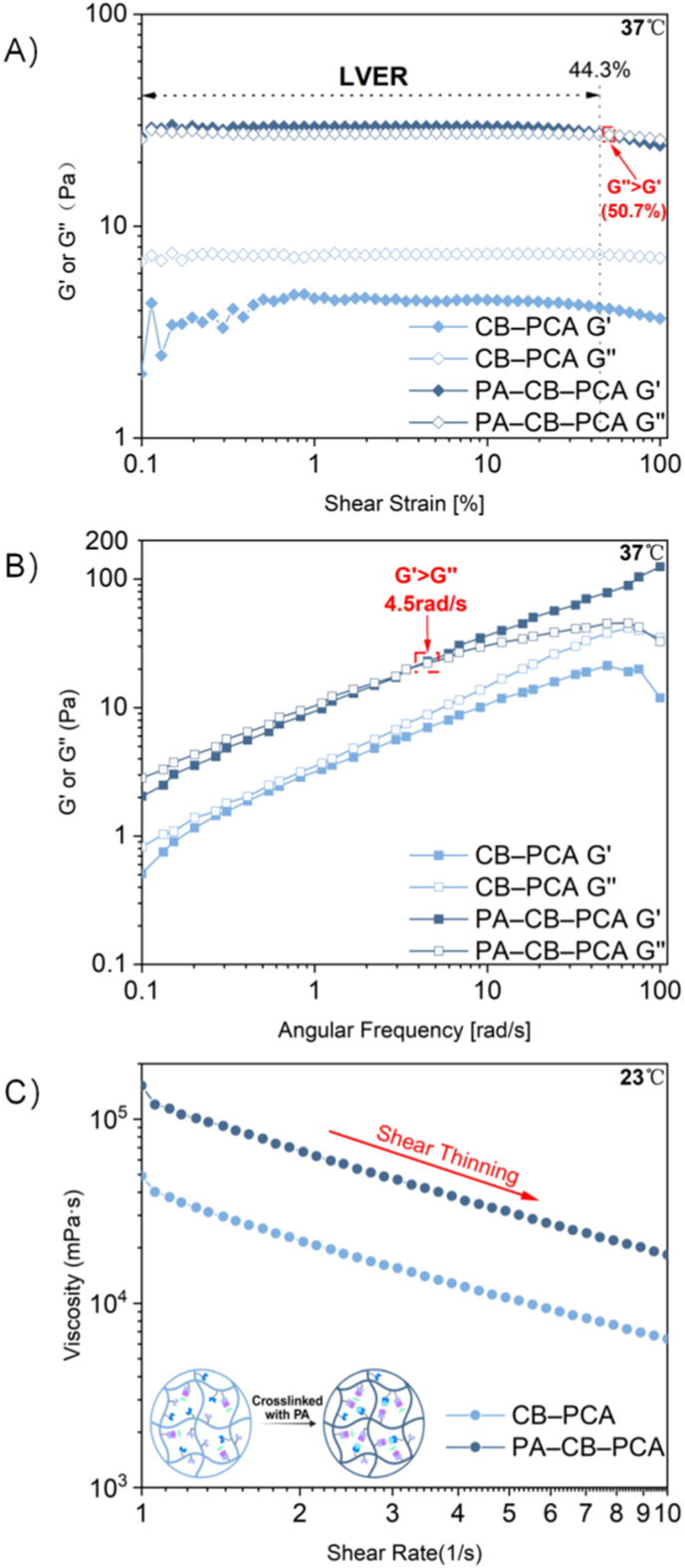
Rheological characterization of the CB–PCA solution and the PA–CB–PCA hydrogel composite. (A) Strain sweep curves illustrating storage modulus (G′) and loss modulus (G″) as a function of oscillatory strain for CB–PCA and PA–CB–PCA at 37 °C; the LVER and moduli crossover have been indicated. (B) Frequency sweep curves showing G′ and G″ for CB–PCA solution and PA–CB–PCA hydrogel composite at 37 °C. (C) Viscosity versus shear rate profiles of CB–PCA and PA–CB–PCA at 23 °C, showing characteristic shear-thinning behavior.

Dynamic strain sweep experiments were conducted to evaluate the mechanical stability, deformation tolerance, and crosslinking-dependent viscoelastic behavior of the DDS after intraperitoneal injection (Fig. 3A). At 37 °C, the noncrosslinked CB–PCA was viscous-dominated, with average G′ and G″ values of 4.18 Pa and 7.32 Pa, respectively, indicating a mechanically weak structure prone to disruption under shear stress. In contrast, the crosslinked PA–CB–PCA hydrogel consistently maintained G′ values higher than G″ across a broad linear viscoelastic region (LVER, 0.09–44.3 %) at low strains (<50.7 %), indicating a robust elastic network capable of maintaining structural integrity and supporting sustained drug release in vivo. Upon increasing the strain to 50.7 %, a crossover of G′ and G″ was observed, followed by a marked decrease in the values of both moduli, indicating shear-induced structural softening. This temporary increase in fluidity suggests that the hydrogel, while structurally stable under physiological conditions, can dynamically reposition itself within the peritoneal cavity rather than remaining fixed at a single location, thereby exhibiting better liver tumor targeting ability [38]. Overall, these characteristics—mechanical stability coupled with shear-responsive mobility—underscore the hydrogel platform's suitability for targeted drug delivery in liver cancer therapy.

Frequency sweep measurements of CB–PCA and PA–CB–PCA were carried out to evaluate the effect of PA crosslinking on hydrogel stability (Fig. 3B). CB–PCA exhibited a slight increase in G′ from 0.51 Pa to 21.27 Pa, but G″ remained higher than G′ across the entire frequency range, indicative of a viscous, liquid-like material lacking mechanical strength. In contrast, PA incorporation resulted in a sharp increase in G′ (2.04 Pa–124.96 Pa), with G″ remaining higher than G′ at low frequencies and the two moduli crossing over at ∼ 4.5 rad/s. This transition from a G″-dominated profile to a G′-dominated profile indicates the formation of an elastic hydrogel network driven by PA crosslinking. Furthermore, the frequency sweep revealed a frequency-dependent transition of PA–CB–PCA from viscous-dominated behavior at low frequencies to elastic-dominated behavior at higher frequencies: the G″-to-G′ crossover occurred at approximately 4.5 rad/s, defining the threshold for hydrogel formation. Because the reported resonant frequencies of the human peritoneum (∼25–50 rad/s) and murine peritoneum (∼553–578 rad/s) are both far above this threshold [39], PA–CB–PCA is expected to maintain its hydrogel state in the peritoneal cavity, thereby withstanding physiological shear and supporting localized, sustained drug release.

The rheological characterization of the hydrogel also involved evaluating its compatibility for intraperitoneal injection under varying temperatures and shear conditions (Fig. 3C). At room temperature (23 °C), all samples exhibited a decrease in viscosity with increasing shear rate, indicating shear-thinning and favorable injectability. At physiological temperature (37 °C), both CB–PCA and PA–CB–PCA exhibited similar shear-dependent behavior (Fig. S6C), which confirmed their structural stability and injectability under physiological conditions.

### 3.4 The hydrogel suppresses cell viability, migration, and colony formation while inducing cell death

We evaluated the anticancer potential of the PA–CB–PCA hydrogel against hepatocellular carcinoma (HepG2) cells using multiple assays: the CCK-8 assay (cell viability), scratch assay (migration), colony formation assay (proliferation), and calcein acetoxymethyl ester (AM) and propidium iodide (PI) double staining (cell survival). This experimental design was based on prior reports that PCA suppresses HCC cell growth. Our findings are consistent with the observations of these studies [40,41].

**Fig. 4:**
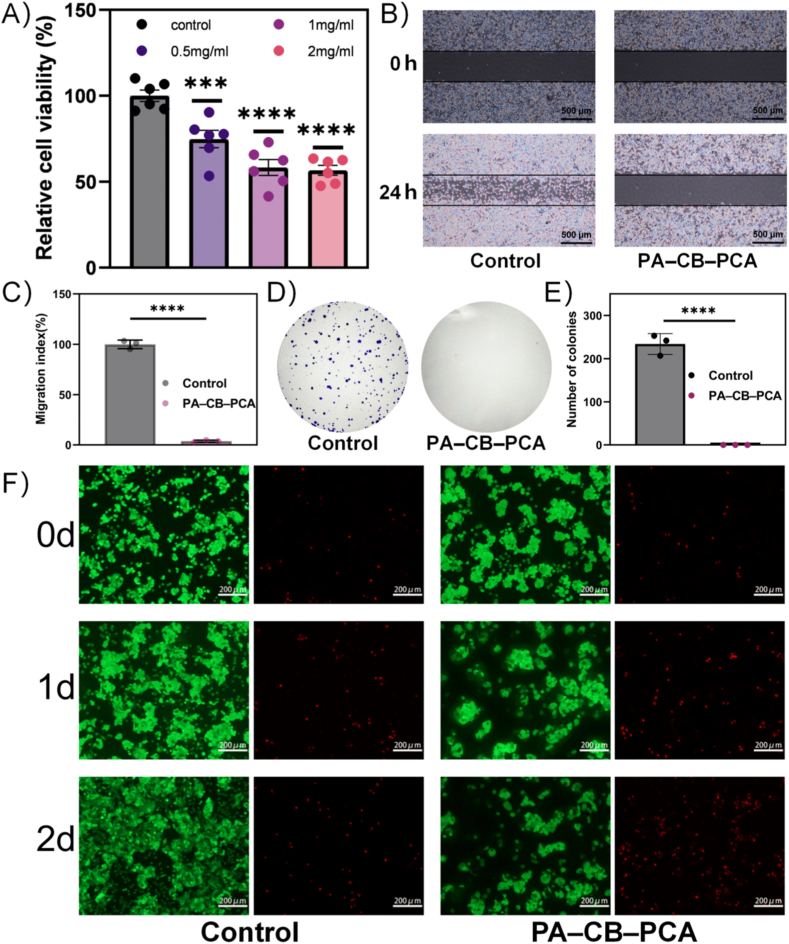
In vitro anticancer effects of PA–CB–PCA on HepG2 cells. (A) CCK-8 assay showing dose-dependent viability reduction. (B, C) Representative images (B) and quantification (C) of cell migration inhibition (scratch assay) after 24 h. (D, E) Colony formation assay images (D) and quantification (E) of proliferation inhibition after 14 days. (F) Live/Dead (Calcein-AM/PI) staining images at different time points. Scale bars: 500 μm in (B) and 200 μm in (F). Data are presented as means ± SD (n = 6 for A; n = 3 for C, E). Statistical significance: ∗∗∗P < 0.001, ∗∗∗∗P < 0.0001.

The CCK-8 assay (Fig. 4A) revealed that at a PA–CB–PCA hydrogel concentration of 0.5 mg/mL, cell viability significantly decreased to approximately 74.9 %; at an elevated concentration of 2 mg/mL, PA–CB–PCA hydrogel reduced cell viability to 56.6 %, indicating a dose-dependent anticancer effect. We further examined the impact of the PA–CB–PCA hydrogel on HepG2 cell migration, given that the invasive and migratory properties of cancer cells are key contributors to tumor metastasis [42,43]. A 24-h scratch assay was performed at cytotoxic concentrations determined in the CCK-8 assay (PA–CB–PCA: 1 mg/mL). As shown in Fig. 4B, the PA–CB–PCA hydrogel significantly inhibited HepG2 cell migration; quantitative analysis (Fig. 4C) revealed a 96.2 % reduction in the PA–CB–PCA group relative to the control. These findings are consistent with the CCK-8 data and further confirm the anticancer potential of the PA–CB–PCA hydrogel.

We conducted a colony formation assay to evaluate the effects of the PA–CB–PCA hydrogel on the proliferative ability of individual cancer cells, as the unlimited proliferative capacity of cancer cells forms the basis for rapid tumor growth and distant metastasis [42,44]. HepG2 cells were treated with PCA or the hydrogel extract for 24 h and then cultured for an additional 14 days. As illustrated in Fig. 4D and E, no visible colonies were observed in the hydrogel-treated groups, indicating significant suppression of cancer cell proliferation.

To evaluate treatment-induced cell death, we carried out a live/dead assay (Calcein-AM/PI double staining), in which viable cells exhibit strong green fluorescence and nonviable cells show red fluorescence (Fig. 4F). Under the same treatment duration, the PA–CB–PCA–treated group displayed markedly increased red fluorescence compared with the control, indicating a higher proportion of dead cells. This effect became more pronounced with prolonged exposure, consistent with the CCK-8 results. Collectively, these findings demonstrate that the PCA-loaded hydrogel effectively suppresses HepG2 cell viability, migration, and proliferation while inducing cell death, underscoring its potential as a promising anti-hepatocellular carcinoma agent.

### 3.5 Acidic tumor microenvironment shifts release kinetics from fickian diffusion to accelerated first-order behavior

**Fig. 5:**
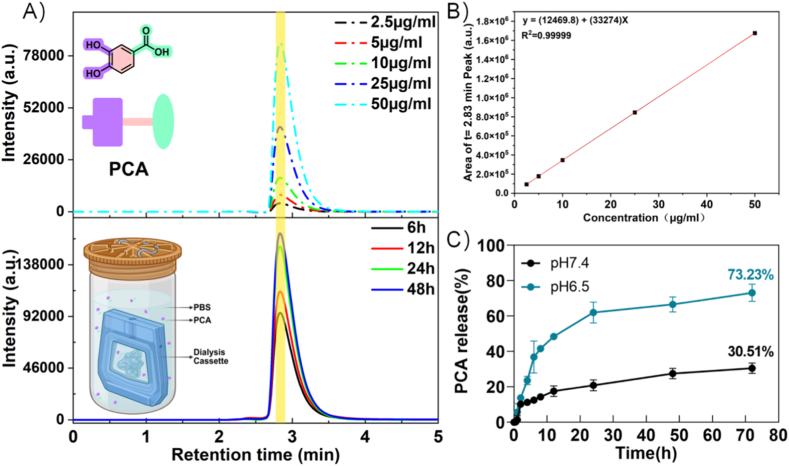
In vitro pH-responsive release of PCA from PA–CB–PCA. (A) HPLC chromatograms of PCA at varying concentrations (top) and PCA released from the hydrogel matrix at different time points (bottom). (B) Standard calibration curve for PCA concentration based on HPLC peak areas. (C) Cumulative release profiles of PCA from PA–CB–PCA in PBS buffer at pH 7.4 and pH 6.5 (37 °C, 100 rpm). Data are presented as the means ± SD (n = 3 for (C)).

To further examine its pH-dependent release profile, the drug-loaded hydrogel was incubated in PBS solution at pH 7.4 (mirroring physiological conditions) and pH 6.5 (mirroring the tumor microenvironment). This experiment was designed to confirm the pH-responsiveness of the reversible boronate ester bonds between PCA and CB, whose hydrolysis under acidic conditions facilitates drug release in the tumor microenvironment. The cumulative PCA release (Fig. 5C), together with HPLC peaks for PA–CB–PCA matching the retention time of free PCA (Fig. 5A, bottom), confirmed the successful release of PCA from the hydrogel. After 72 h, the cumulative release of PCA reached 30.51 % at pH 7.4 and 73.23 % at pH 6.5.

Enhanced PCA release under acidic conditions arises from the hydrolysis of two sets of pH-sensitive bonds: (i) the PCA–CB boronate ester bonds, whose cleavage directly releases PCA [45] and (ii) PA–CB Schiff base and boronate ester bonds [46,47], whose cleavage destabilizes the hydrogel network and relaxes the polymer chains, thereby further accelerating PCA release.

To investigate the in vitro drug release behavior of the PA–CB–PCA drug–hydrogel composite, a quantitative analysis of PCA was performed using HPLC. PCA appeared as a distinct peak at a retention time of 2.83 min (Fig. 5A, top); a standard calibration curve was subsequently generated from the peak intensity (Fig. 5B).

To further characterize the underlying release mechanism, we evaluated the drug-release kinetics of PCA from PA–CB–PCA. The cumulative release profiles at pH 6.5 (responsive condition) and pH 7.4 were fitted to zero-order, first-order, Higuchi, and Korsmeyer–Peppas models (Fig. S7). Table 2 summarizes the kinetic rate constants and correlation coefficients (R^2^) for each model under both pH conditions, allowing comparison of their fit to the release mechanism. In the physiological environment (pH 7.4), the Korsmeyer–Peppas model provided the best fit (R^2^ = 0.9459), with a release exponent n < 0.45, indicating that PCA release is primarily governed by Fickian diffusion, i.e., diffusion driven by the concentration gradient [48]. Conversely, under tumor microenvironment conditions (pH 6.5), the first-order kinetic model outperformed other models, exhibiting the highest correlation coefficient (R^2^ = 0.9910). This behavior can be attributed to hydrogel breakdown and PCA dissolution occurring concurrently in the acidic environment, leading to accelerated release [49]. Mechanistically, such a pH-responsive shift in kinetics arises because, in chitosan-based hydrogels, protonation of amino groups (-NH_2_ to -NH_3_^+^) in acidic environments [50,51] induces electrostatic repulsion, in turn causing polymer chain extension and rapid hydrogel swelling. The resulting enlargement of mesh pores expedites drug dissolution and diffusion. Together, these features highlight the PA–CB–PCA composite as an optimized system for tumor therapy.

**Table 2:** Kinetic parameters and correlation coefficients (R^2^) for different kinetic models of PCA release from PA–CB–PCA at pH 7.4 and pH 6.5.

	Zero-order kinetics		First-order kinetics		Higuchi model		Korsmeyer-Peppas model		
	K	R^2^	K	R^2^	K	R^2^	K	R^2^	n
pH7.4	0.3888	0.7393	0.0943	0.9334	3.7514	0.9221	5.8517	0.9459	0.3969
pH6.5	0.9546	0.6497	0.1087	0.9910	9.5195	0.8763	15.5636	0.9122	0.3842

### 3.6 SA-rich HCC cells and tumors preferentially accumulate CB-based hydrogels

**Fig. 6:**
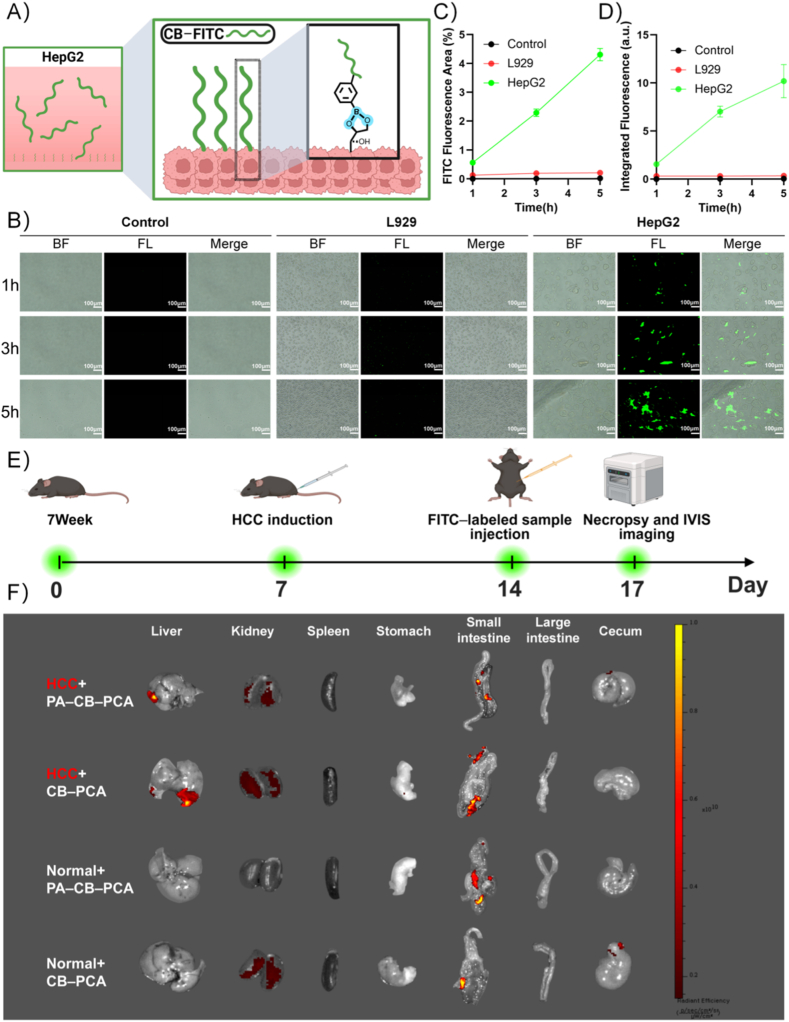
Tumor-specific cellular uptake and in vivo targeting of BA-modified hydrogels. (A) Schematic mechanism of SA-mediated uptake by HepG2 cells. (B) Fluorescence images displaying time-dependent uptake (1, 3, and 5 h) in control, L929, and HepG2 cells; columns correspond to bright field (BF), fluorescence (FL), and merged (Merge) channels. Scale bar = 100 μm. (C, D) Quantitative analysis of (C) fluorescence area and (D) intensity over time. (E) Experimental timeline: 7-week-old mice arrived on Day 0, followed by HCC induction (Day 7), hydrogel injection (Day 14), and necropsy/imaging (Day 17). (F) IVIS bioluminescence images of PA–CB–PCA and CB–PCA biodistribution in HCC-bearing and normal mice. Fluorescence accumulation is displayed using a radiant efficiency color scale (p/sec/cm^2^/sr)/(μW/cm^2^). Data are presented as the means ± SD (n = 3 for (C), (D) and (F)). (For interpretation of the references to color in this figure legend, the reader is referred to the Web version of this article.)

To further validate the specificity of the material toward cancer cells, L929 (non-tumor cells) and HepG2 (hepatocellular carcinoma cells) were cultured in the presence of fluorescein-isothiocyanate-labeled CB (CB–FITC). Acting as the specific recognition target for CB, sialic acid (SA) is typically exposed at glycan terminals and overexpressed on the surface of various cancer cells, serving as a sensitive diagnostic biomarker [52,53]. Significantly, a study by Liang et al. indicated that the overall sialylation level on the surface of HepG2 cells is approximately 20–40 times higher than that of normal hepatocyte lines (e.g., HL-7702) [54]. Given this significant disparity, exploiting the specific affinity between phenylboronic acid and sialic acid has become a widely established strategy for designing targeted delivery systems against hepatocellular carcinoma [55]. Based on this mechanism, we hypothesized that the overexpressed sialic acid residues on the HepG2 surface should form stable boronate ester bonds with the boronic acid groups in CB (Fig. 6A), thereby specifically mediating the efficient uptake of the material by cancer cells. To molecularly validate this key binding mechanism, CB was reacted with sialic acid under weakly acidic conditions simulating the tumor microenvironment (pH 6.5–7.0) [56,57]; subsequent ^1^H NMR and FT-IR analyses confirmed the formation of boronate ester bonds (Fig. S8B and S8C). As shown in Fig. 6B, the fluorescence intensity and area of FITC in HepG2 cells increased markedly over time, whereas only minimal signals were observed in L929 cells and the control group. After 5 h of incubation, the fluorescence area in HepG2 cells reached 4.3 %—approximately 21 times higher than in L929 cells (Fig. 6C)—and the fluorescence intensity reached 10.2 a.u., approximately 29 times greater than that of L929 cells (Fig. 6D). Notably, this distinct uptake difference (approx. 21–29-fold) is highly consistent with the quantitative data reported by Liang et al., which showed that sialylation levels on HepG2 cells are 20–40 times higher than those on normal cells [54]. This consistency strongly corroborates our design rationale that the high targeting specificity of CB for HepG2 cells stems primarily from the specific interaction between phenylboronic acid and the overexpressed sialic acid.

Importantly, our system differs from conventional chitosan hydrogels that rely primarily on passive accumulation, in that drug release may be coupled to renewed tumor-associated binding. We describe this as an “acid-triggered ligand re-exposure” mechanism: under mildly acidic tumor-microenvironment conditions, decomplexation of PCA–boronate esters releases the payload while potentially restoring available boronic-acid sites on the scaffold that were partially occupied during loading. The re-exposed boronic-acid ligands may re-engage SA residues on HCC cells, thereby supporting local association after release. Collectively, this provides a mechanistic rationale for coupling pH-triggered release with tumor-associated retention.

To confirm whether the targeting capability of the DDS is maintained in vivo, FITC-CB was administered intraperitoneally in healthy and HCC-bearing mice. Three days after injection, the mice were euthanized and the biodistribution of the hydrogel was analyzed (Fig. 6E). As illustrated in Fig. 6F, strong fluorescence signals were observed in the liver of the HCC group, indicating significant tumor-targeted accumulation of both PA–CB–PCA and CB–PCA; however, no notable accumulation was detected in the liver samples of the normal control group. Additionally, mice administered CB–PCA exhibited a higher renal fluorescence intensity than those administered PA–CB–PCA, suggesting that PA incorporation slows hydrogel metabolism in vivo, thereby enhancing structural stability and prolonging drug release. These findings confirm the tumor-targeting ability of the hydrogel in vivo and demonstrate that PA incorporation improves the hydrogel stability and sustains drug release, highlighting potential of the DDS for long-term therapeutic use.

### 3.7 Intraperitoneal injection of PA–CB–PCA reduces tumor burden and restores liver function

**Fig. 7:**
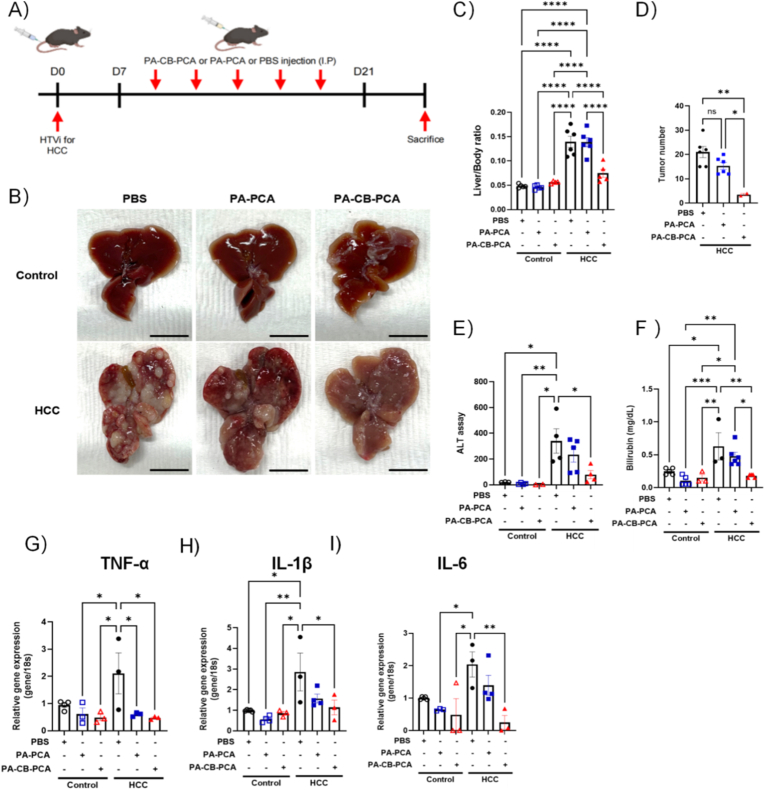
In vivo therapeutic efficacy of PA–CB–PCA in an HCC mouse model. (A) Experimental timeline: HCC induction (HTVI) in 6-week-old mice, followed by treatments (PBS, PA–PCA or PA–CB–PCA) every 3 days from Day 7 until sacrifice on Day 21. (B) Representative macroscopic liver images. Scale bar = 1 cm. (C, D) Analysis of (C) liver-to-body-weight ratio and (D) number of visible tumor nodules. (E, F) Serum levels of (E) ALT and (F) direct bilirubin. (G–I) Relative mRNA expression of inflammatory cytokines (G) TNF-α, (H) IL-1β, and (I) IL-6 in liver tissue (Data represent relative mRNA expression, normalized to housekeeping gene 18S). Data are presented as mean ± SEM. ∗P < 0.05, ∗∗P < 0.01, ∗∗∗P < 0.001, ∗∗∗∗P < 0.0001; ns, not significant.

To evaluate the therapeutic efficacy of PA–CB–PCA in vivo, an HCC model was established in C57BL/6J mice via hydrodynamic tail vein injection (HTVI) of oncogene (NRAS) and tumor suppressor (Pten and Tp53) plasmids. Quantitative real-time polymerase chain reaction (qRT-PCR) detected the anticipated alterations in liver gene expression, confirming the successful induction of the tumor (Fig. S9). The HCC-induced mice were administered PBS, PA–PCA, or PA–CB–PCA via intraperitoneal injection every three days for three weeks (Fig. 7A). Macroscopic examination of harvested livers revealed markedly reduced tumor burden and overall pathological changes in PA–CB–PCA-treated HCC mice compared to the PBS controls (Fig. 7B). Quantitative analysis confirmed significant decreases in both the liver-to-body-weight ratio (Fig. 7C) and the number of visible tumor nodules per liver in PA–CB–PCA-treated mice (Fig. 7D). PA–CB–PCA administration also lowered absolute liver weight (Fig. S10), further supporting the observed reduction in tumor burden and associated liver hypertrophy. While PA–PCA showed modest therapeutic effects, PA–CB–PCA was markedly more effective in reducing tumor numbers. This enhanced efficacy underscores the critical role for the CB scaffold in enhancing therapeutic outcomes, potentially through improved drug delivery and bioavailability.

To assess liver function, the serum levels of hepatic injury biomarkers were measured. Serum alanine aminotransferase (ALT), direct bilirubin, and total bilirubin levels, significantly elevated in HCC mice, were markedly reduced upon PA–CB–PCA treatment (Fig. 7E and F; Fig. S11). These findings suggest that PA–CB–PCA helps preserve liver integrity and improve hepatic function during HCC.

Expression of inflammatory cytokines (TNF-α, IL-1β, and IL-6), known to drive HCC progression, was assessed using qRT-PCR. These cytokines were markedly upregulated in HCC-induced livers (Fig. 7G-I). PA–CB–PCA treatment significantly suppressed their elevated mRNA levels, demonstrating potent anti-inflammatory effects within the liver microenvironment. In addition, PA-CB-PCA exhibited dose-dependent antioxidant activity in the DPPH radical-scavenging assay in vitro (Fig. S12), consistent with the anti-inflammatory phenotype observed in vivo [58,59].

### 3.8 Post-treatment fibrosis and tissue damage are significantly attenuated

**Fig. 8:**
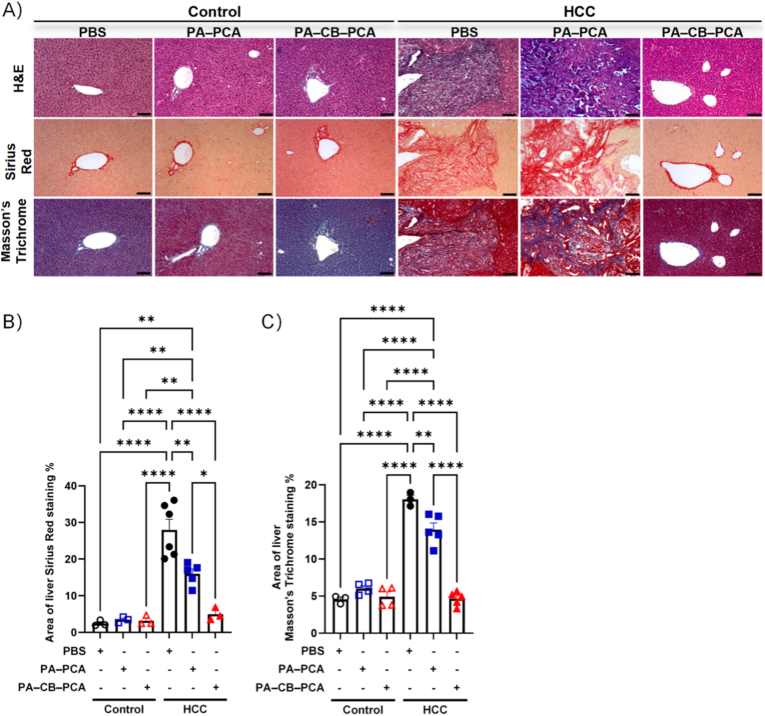
Histological evaluation of liver damage and fibrosis in PA–CB–PCA-treated HCC mice. (A) Representative images of H&E−, Sirius-Red-, and Masson's-trichrome-stained liver sections from control and HCC mice treated with PBS, PA–PCA, or PA–CB–PCA. Scale Bar = 100 μm. (B) Quantification of Sirius-Red-positive area (%) in liver tissues from control and HCC groups. (C) Quantification of Masson's-trichrome-positive area (%) in liver tissues from control and HCC groups. Data are presented as mean ± SEM. ∗P < 0.05, ∗∗P < 0.01, ∗∗∗∗P < 0.0001. (For interpretation of the references to color in this figure legend, the reader is referred to the Web version of this article.)

Histopathological analysis was performed to assess the extent of liver damage and fibrosis. Hematoxylin and eosin (H&E) staining revealed severe architectural damage and extensive tumor infiltration in HCC livers, while PA–CB–PCA treated groups exhibited significantly preserved structure with reduced infiltration (Fig. 8A). Collagen deposition, a hallmark of fibrosis, was evaluated using Sirius Red and Masson's trichrome staining. HCC livers exhibited extensive fibrosis, with markedly increased staining areas for Sirius Red (Fig. 8B) and Masson's trichrome (Fig. 8C). PA–CB–PCA treatment significantly reduced collagen deposition, leading to a notable attenuation of liver fibrosis.

Taken together, these results demonstrate that PA–CB–PCA, a pH-responsive drug–hydrogel complex that enables controlled release and targeted delivery of plant-derived polyphenols, exerts potent antitumor, anti-inflammatory, and antifibrotic effects, thus showing multifaceted therapeutic activity against HCC. These findings highlight our system as an effective pH-responsive DDS for HCC.

### 3.9 Synergistic effects of PCA and DDS, and study limitations

The therapeutic effects observed here are consistent with the reported anti-inflammatory and antioxidant activities of PCA [40,60]. Prior studies have linked PCA-associated anti-inflammatory phenotypes to modulation of NF-κB signaling, a central regulator of inflammatory responses [[61], [62], [63]]. In our study, qRT-PCR analysis showed reduced hepatic expression of TNF-α, IL-6, and IL-1β following treatment (Fig. 7G–I), which is consistent with attenuated inflammatory signaling and aligns with an NF-κB–associated mechanism proposed in the literature [62,63]. Because NF-κB is also implicated in the interface between liver injury, fibrosis, and hepatocarcinogenesis [63], this framework provides a unified interpretation for the concurrent decreases in inflammatory cytokines, fibrosis-related readouts, and tumor burden.

Importantly, the PA–PCA group (drug without carrier) showed measurable, yet comparatively modest, improvements relative to the untreated control (Fig. 7, Fig. 8), supporting a contribution from PCA itself. However, free PCA is reported to undergo rapid in vivo clearance/short systemic exposure [24], which may limit the durability of its pharmacological effects. In the PA–CB–PCA group, the hydrogel formulation likely enhances local drug exposure through tumor-associated accumulation (Fig. 6) and pH-responsive release (Fig. 5), thereby amplifying the magnitude of PCA-linked anti-inflammatory and anti-fibrotic responses observed in vivo. Together, these results support a model in which PCA provides the primary bioactive mechanism, while DDS features increase effective exposure at the disease site to strengthen the overall therapeutic outcome.

While this study demonstrates the therapeutic potential of PA–CB–PCA, the current conclusions are based on in vitro testing in a single HCC cell line (HepG2) and in vivo evaluation in one HTVI-driven HCC model. Direct benchmarking against first-line systemic therapies (e.g., sorafenib or lenvatinib) was not included in the present work. In addition, safety assessment was limited to the study timeframe and dosing schedule, and longer-term toxicity and immune responses under extended, repeated dosing remain to be further characterized. Intraperitoneal administration was used here as a controlled preclinical route to assess depot formation, tumor-selective accumulation, and pH-responsive release, and should be interpreted in that context.

## 4 Conclusion

HCC remains a global challenge with poor prognosis. In this study, we present an injectable, pH-responsive hydrogel loaded with PCA (PA–CB–PCA), which enables targeted recognition, sustained drug delivery, and acidic-triggered release. The system demonstrated superior efficacy compared to free PCA. The formulation achieved a near-complete reduction in HepG2 cell growth and migration in vitro, while markedly enhancing apoptosis and reducing tumor burden in vivo—as reflected by decreased nodule counts, liver-to-body weight ratio, fibrosis, serum ALT/bilirubin levels, and proinflammatory cytokine (IL-6, IL-1β, and TNF-α) expression. These results highlight the system's capacity to address key limitations of PCA—metabolic instability and limited tumor specificity—by enabling sustained release and targeted accumulation. Overall, this hydrogel framework is a versatile platform for the targeted delivery of polyphenolic agents, with potential therapeutic applications against HCC and other cancers.

## CRediT authorship contribution statement

**Weiqiang Hao:** Writing – review & editing, Writing – original draft, Visualization, Validation, Methodology, Investigation, Formal analysis, Data curation, Conceptualization. **Hyeon Ji Kim:** Writing – review & editing, Writing – original draft, Validation, Investigation, Formal analysis, Data curation. **Jumi Kang:** Investigation. **Bongkyun Kang:** Investigation. **Seoyeon Park:** Investigation. **Yuejin Kim:** Data curation, Formal analysis. **Eunjeong Kim:** Writing – review & editing, Supervision, Resources, Project administration, Methodology, Funding acquisition, Conceptualization. **Kyueui Lee:** Writing – review & editing, Supervision, Resources, Project administration, Methodology, Funding acquisition, Conceptualization.

## Declaration of competing interest

The authors declare the following financial interests/personal relationships which may be considered as potential competing interests: Kyueui Lee and Weiqiang Hao has patent pending to Kyungpook National University. If there are other authors, they declare that they have no known competing financial interests or personal relationships that could have appeared to influence the work reported in this paper.

## Data Availability

No data was used for the research described in the article.
