# Supplementary material for: Sialic acid–guided spatiotemporal hydrogel therapy for liver cancer
Source: Mater Today Bio. 2026 Jan 8;36:102784. doi: 10.1016/j.mtbio.2026.102784 (PMC12825078; doi:10.1016/j.mtbio.2026.102784)
Supplement: Multimedia component 1 [file mmc1.pdf]

Sialic Acid – Guided Spatiotemporal Hydrogel Therapy for Liver Cancer

Weiqiang Hao[a,‡], Hyeon Ji Kim[b,e,f,‡], Jumi Kang[a], Bongkyun Kang[c], Seoyeon Park[d], Yuejin Kim[a], Eunjeong Kim[b,e,f,*], Kyueui Lee[a,c,g,*]

[a]*Department of Chemistry, Kyungpook National University, Daegu 41566, South Korea*

[b]*Department of Biology, College of Natural Sciences, Kyungpook National University, Daegu 41566, Republic of Korea*

[c]*KNU Institute of Basic Sciences and KNU G-LAMP Project Group, Kyungpook National University, Daegu 41566, South Korea*

[d]*School of Life Science and Biotechnology, Kyungpook National University, Daegu 41566, South Korea*

[e]*School of Life Sciences, BK21 FOUR KNU Creative BioResearch Group, Kyungpook National University, Daegu, 41566, Republic of Korea*

[f]*Center for Genome Engineering, Institute for Basic Science, Daejeon 34126, Republic of Korea*

[g]*Biomedical Research Institute, Kyungpook National University Hospital, Daegu 41940, South Korea*

* Corresponding author.

E-mail address: kyueui@knu.ac.kr (K. Lee), eunjkim@knu.ac.kr (E. Kim).

‡Weiqiang Hao and Hyeon Ji Kim contributed equally to this work.

TABLE OF CONTENTS

TABLE OF CONTENTS 2
Figure S1 3
Figure S2 4
Figure S3 5
Figure S4 6
Figure S5 7
Figure S6 8
Figure S7 10
Figure S8 10
Figure S9 12
Figure S10 13
Figure S11 13
Figure S12 14
REFERENCES 15

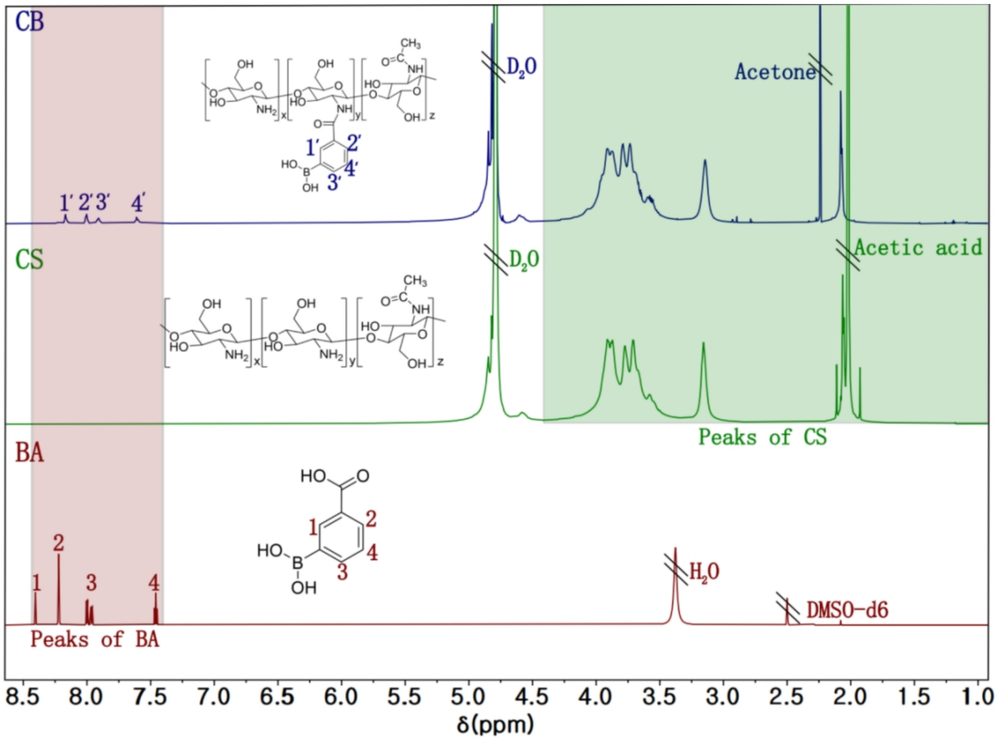

Figure S1. ^{1}H NMR analysis of 3-boronobenzoic acid (BA), chitosan (CS), and chitosan–boronobenzoic acid (CB).

As shown in Figure S1, proton nuclear magnetic resonance spectroscopy (^{1}H NMR) confirmed the successful grafting of 3-boronobenzoic acid (BA) onto chitosan (CS), resulting in the formation of chitosan–boronobenzoic acid (CB).[1-3] The spectrum of CB exhibits characteristic signals corresponding to both CS and BA, indicating the formation of a covalent bond between the carboxyl group of BA and the amino group of CS. Specifically, the spectrum of CS displays proton signals of the glucosamine units in the range of 1.0-5.5 ppm.[2] In contrast, the spectrum of BA shows aromatic proton peaks in the range 7.5–8.5 ppm (labeled 1–4), corresponding to a meta-substituted benzene ring.[4-5] Notably, the CB spectrum retains the backbone signals of CS (1.0–5.5 ppm) while also exhibiting the aromatic proton peaks derived from BA (7.5–8.5 ppm, labeled 1′–4′). This confirms the successful incorporation of the BA aromatic ring into the CS backbone.[5]

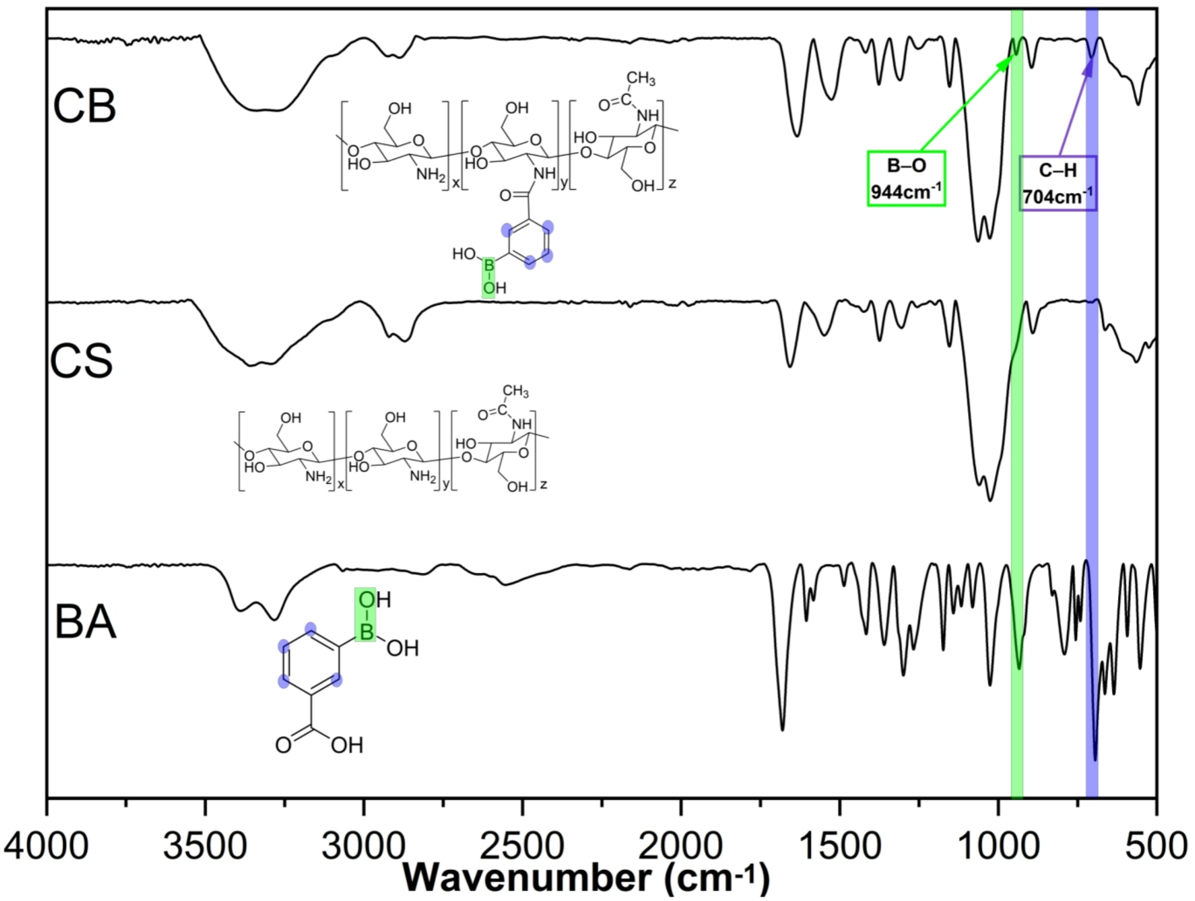

Figure S2. FT-IR spectra of BA, CS, and CB.

As shown in Figure S2, Fourier-transform infrared spectroscopy (FT-IR) further confirms the successful synthesis of CB. After the reaction between BA and CS, the FT-IR spectrum of CB exhibits two prominent new absorption peaks: one at 944 cm^{-1}, corresponding to B–O stretching vibrations,[6-8] and another at 704 cm^{-1}, attributed to the out-of-plane bending vibrations of aromatic C–H.[9-11] These bonds are absent in the original CS spectrum. Moreover, while native CS was only soluble in acidic solutions,[12-14] the synthesized CB exhibited excellent solubility in phosphate-buffered saline (PBS) at pH 7.4. This improved solubility, together with the observed spectral changes, provides strong evidence toward the successful synthesis of CB.

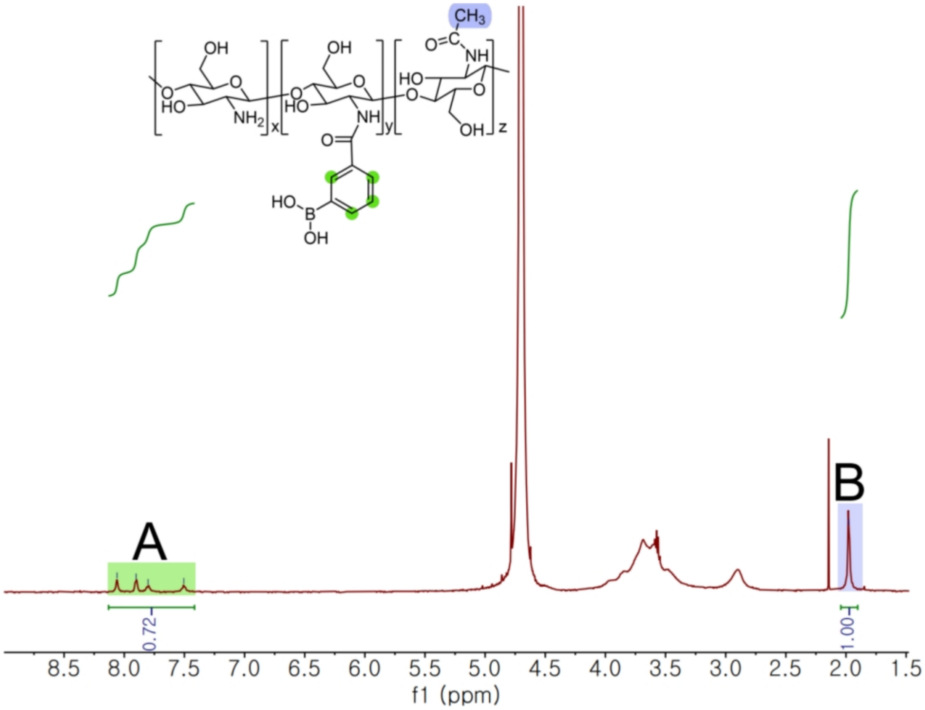

Figure S3. Calculation of the degree of substitution (DOS) of BA.

The CS used in this study had a deacetylation degree of ~80% (range: 75–85%). Therefore, the corresponding degree of acetylation (DA) was estimated to be around 20%. Based on this, the degree of substitution (DOS) of BA on CS was calculated using the following equation:

$$\begin{aligned} \text{DOS (\%)} &= \frac{(I_A/4)}{(I_B/3)/\text{DA}} \times 100\% \\ &= \frac{(0.72/4)}{(1/3)/20\%} \times 100\% \qquad \text{(Equation S1)} \\ &= 10.8\% \end{aligned}$$

where I_A is the integral area of the aromatic proton region corresponding to the benzene ring of BA, with 4 being the number of aromatic protons. I_B is the integral area of the methyl protons in the acetyl group, and 3 is the number of protons contributing to this peak. DA represents the degree of acetylation of CS. In this experiment, the DOS of the synthesized CB was determined to be 10.8%.

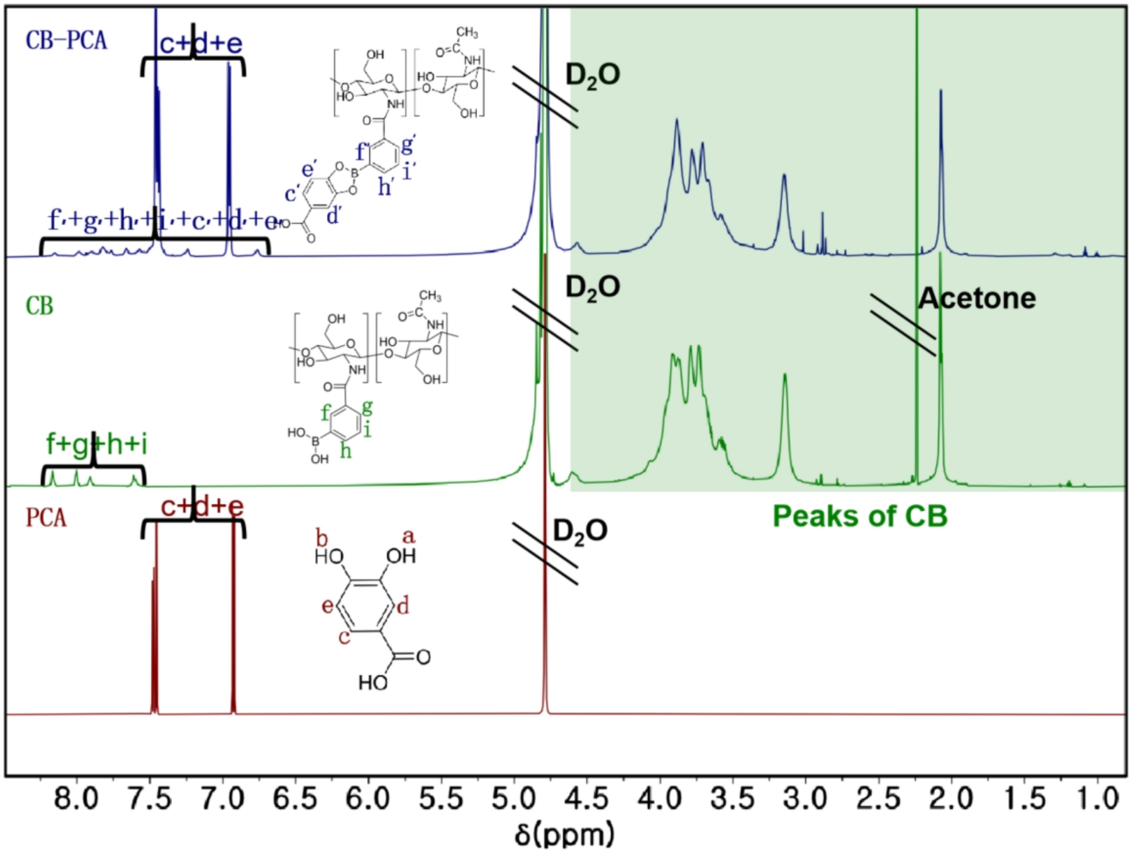

Figure S4. ^{1}H NMR analysis of protocatechuic acid (PCA), CB, and CB–PCA

The successful loading of protocatechuic acid (PCA) onto CB was confirmed by the ^{1}H NMR spectrum in Figure S4. Upon mixing CB with PCA, the original proton signals of CB (peaks f–i) and PCA (peaks c, d, and e) underwent significant chemical shifts, manifesting as new peaks (f'–i' and c'–d'–e') in the ^{1}H NMR spectrum of the CB–PCA complex. These shifts indicate effective binding interactions between CB and PCA, likely driven by the formation of boronate ester bonds. However, the spectrum of CB−PCA also reveals unshifted peaks corresponding to free PCA (c, d, and e), suggesting that a portion of PCA remains unbound. This unbound PCA is likely incorporated into the hydrogel matrix through noncovalent interactions (such as hydrogen bonding and π–π stacking).

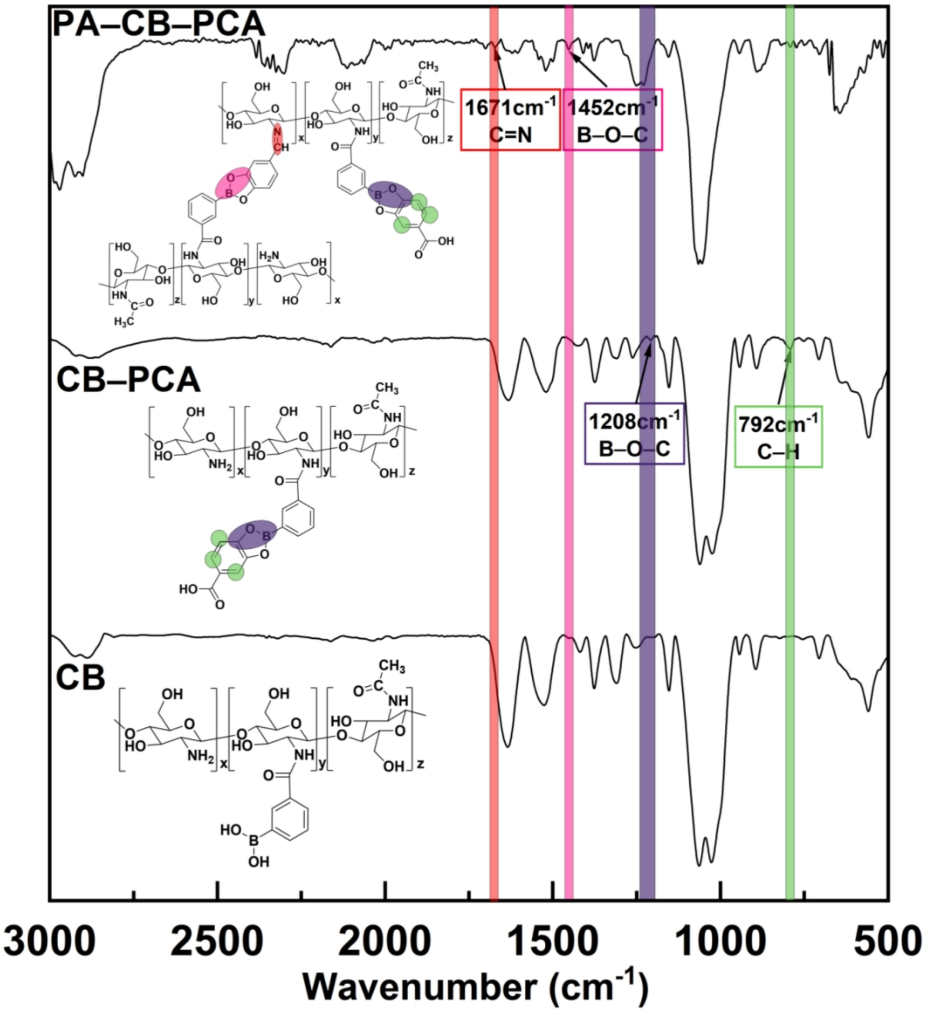

Figure S5. FT-IR spectra of CB, CB–PCA, and PA–CB–PCA

FT-IR analysis (Figure S5) further confirmed the interaction between CB and PCA. In the spectrum of CB–PCA, an absorption peak characteristic of B–O–C stretching vibrations appeared at 1208 cm^{-1},[15] confirming the formation of boronate ester bonds. Additionally, a new absorption peak emerged at 792 cm^{-1}, attributed to the out-of-plane bending vibrations of C–H on the PCA benzene ring,[9-11] which further supports the successful loading of PCA onto CB *via* boronate ester complexation. To prolong *in vivo* retention and prevent rapid clearance after injection, protocatechualdehyde (PA) was introduced to crosslink CB–PCA through Schiff-base reactions and boronate ester bonds, yielding a three-dimensional hydrogel structure (PA–CB–

PCA). The FT-IR spectrum shows a prominent absorption peak at 1671 cm^{-1},[16-17] corresponding to the stretching vibrations of the C=N bond. This provides clear evidence of the successful introduction of imine groups, indicating that PA reacts with CB–PCA through Schiff-base linkage formation. Furthermore, a characteristic absorption peak corresponding to B–O–C vibrations was observed at 1452 cm^{-1},[18] further confirming the formation of boronate esters. In summary, these results indicate that PA effectively crosslinks the CB–PCA network, significantly enhancing the structural stability of the hydrogel.

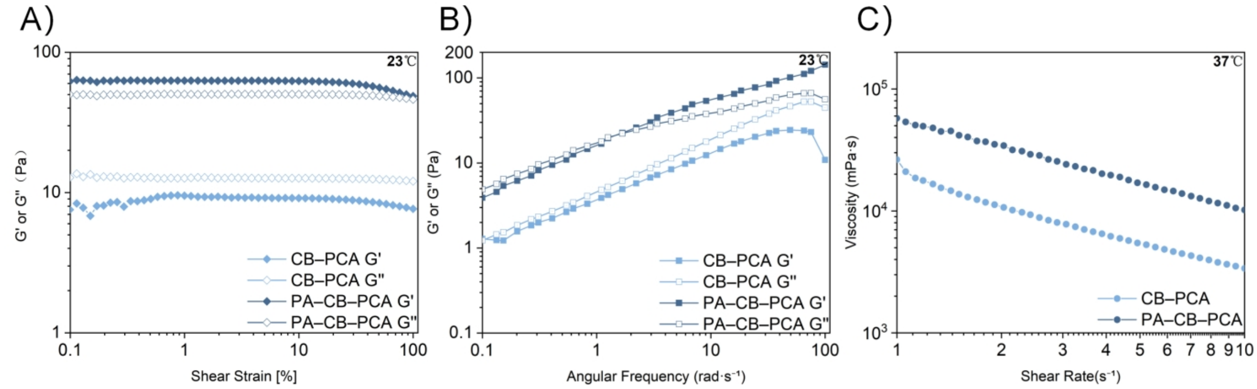

Figure S6. Rheological characterization of CB, CB–PCA, and PA–CB–PCA. (**A**) Strain sweep illustrating storage modulus (G′) and loss modulus (G″) as a function of shear strain for CB–PCA and PA–CB–PCA at 23 °C, highlighting the linear viscoelastic region and moduli crossover. (**B**) Frequency sweep showing G′ and G″ for the CB–PCA solution and the PA–CB–PCA hydrogel at 23 °C. (**C**) Viscosity as a function of shear rate for CB–PCA and PA–CB–PCA at 37 °C, demonstrating shear-thinning behavior.

pH6.5

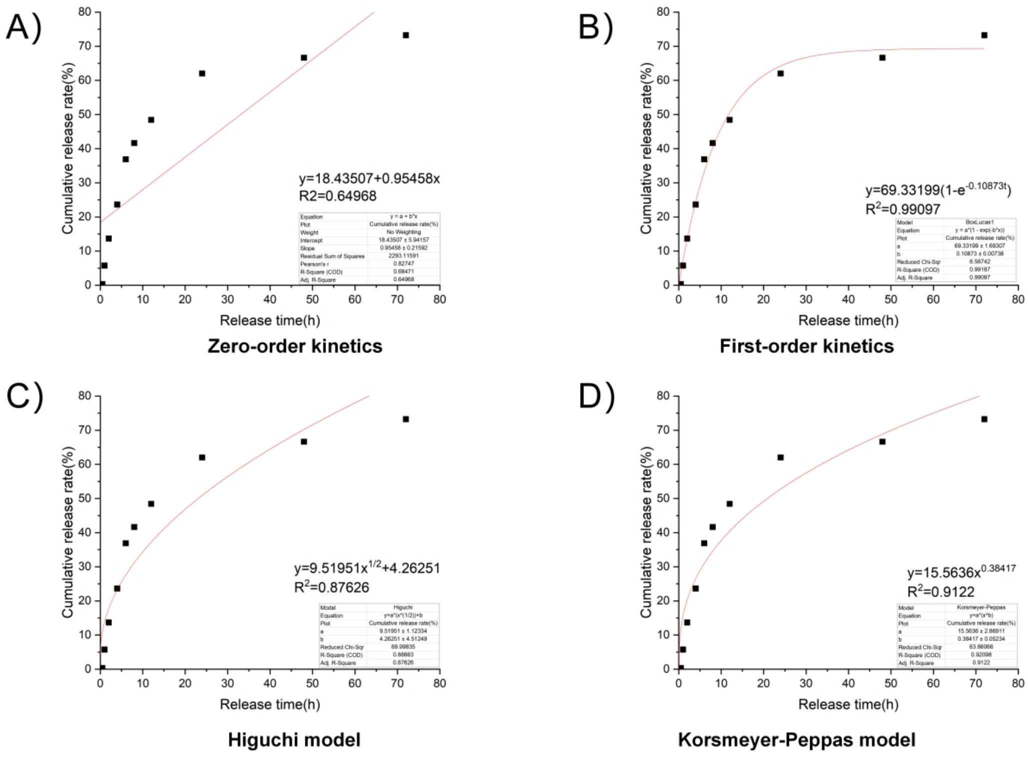
A)
y=18.43507+0.95458x
R2=0.64968
Cumulative release rate(%)
Release time(h)
Zero-order kinetics
B)
y=69.33199(1-e^-0.10873t)
R²=0.99097
Cumulative release rate(%)
Release time(h)
First-order kinetics
C)
y=9.51951x^1/2+4.26251
R²=0.87626
Cumulative release rate(%)
Release time(h)
Higuchi model
D)
y=15.5636x^0.38417
R²=0.9122
Cumulative release rate(%)
Release time(h)
Korsmeyer-Peppas model

pH7.4

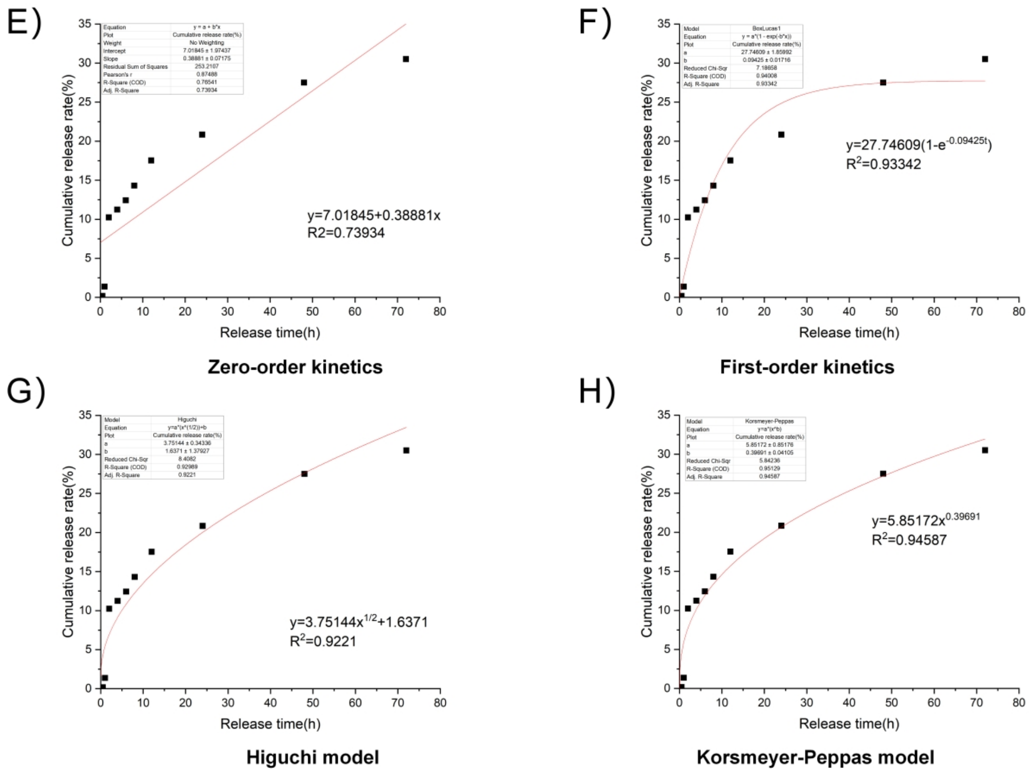
E)
y=7.01845+0.38881x
R2=0.73934
Cumulative release rate(%)
Release time(h)
Zero-order kinetics
F)
y=27.74609(1-e^-0.09425t)
R²=0.93342
Cumulative release rate(%)
Release time(h)
First-order kinetics
G)
y=3.75144x^1/2+1.6371
R²=0.9221
Cumulative release rate(%)
Release time(h)
Higuchi model
H)
y=5.85172x^0.39691
R²=0.94587
Cumulative release rate(%)
Release time(h)
Korsmeyer-Peppas model

Figure S7. Fitting of cumulative PCA release from the PA–CB–PCA hydrogel at pH 6.5 to (**A**) zero-order kinetics, (**B**) first-order kinetics, (**C**) the Higuchi model, and (**D**) the Korsmeyer–Peppas model; Fitting of cumulative PCA release from the PA–CB–PCA hydrogel at pH 7.4 to (**E**) zero-order kinetics, (**F**) first-order kinetics, (**G**) the Higuchi model, and (**H**) the Korsmeyer–Peppas model.

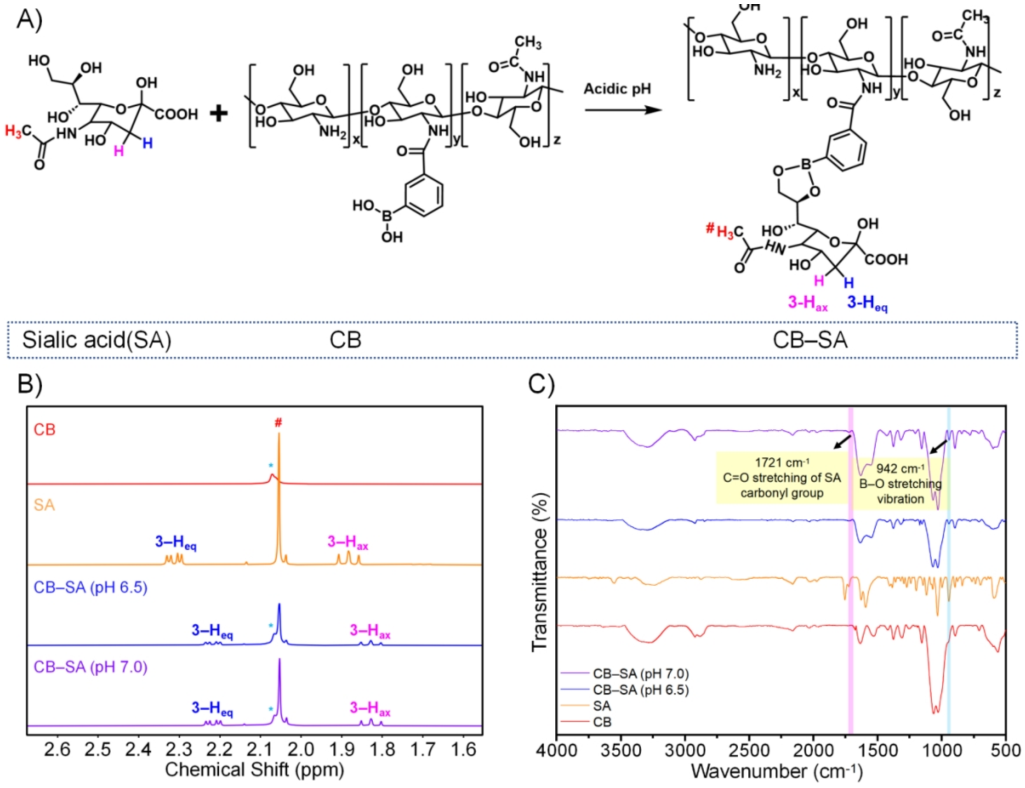

Figure S8. (**A**) Schematic illustration of the conjugation reaction between CB and sialic acid (SA) via boronate ester bond formation in acidic pH. (**B**) ^{1}H NMR spectra of CB, SA, and CB–SA prepared at pH 6.5 and pH 7.0, highlighting upfield shifts in the peaks corresponding to SA protons (e.g., 3-Heq from 2.33 to 2.23 ppm and 3-Hax from 1.91 to 1.85 ppm) and the N-acetyl methyl group at 2.05 ppm. (**C**) FT-IR spectra of CB, SA, and CB–SA prepared at pH 6.5 and pH 7.0, showing characteristic peaks at 1721 cm^{-1} (C=O stretching of the SA carboxyl group) and 942 cm^{-1} (B–O stretching of the boronate ester linkage).

Considering the mildly acidic pH (~6.8) characteristic of the tumor microenvironment, we selected buffer solutions set to pH 6.5 and pH 7.0 to facilitate the reaction between CB and SA. The mixtures were incubated at 37°C for 4 h to simulate the tumor-like conditions, enabling the evaluation of boronic-acid-mediated interactions between CB and SA.

Upon completion of the reaction, the products were dialyzed and subsequently characterized by ^{1}H NMR spectroscopy. As illustrated in Figure S8B, the proton signals corresponding to SA exhibited a discernible upfield shift after conjugation with CB, indicative of successful covalent bonding. Specifically, the equatorial proton (3-Heq) and axial proton (3-Hax) of SA (Figure S8A) shifted from 2.33 ppm to 2.23 ppm and 1.91 ppm to 1.85 ppm, respectively. Additionally, the characteristic resonance of the N-acetyl methyl group in the SA moiety emerged at 2.05 ppm, further corroborating the successful conjugation and structural integrity of the product.[19-20]

To further substantiate the formation of the boronate ester linkage between CB and SA, FT-IR spectroscopy was performed. As shown in Figure S8C, the absorption band observed at 1721 cm^{-1} was attributed to the C=O stretching vibrations of the carboxyl group in SA,[21] indicating the preservation of its functional groups even after conjugation. Moreover, a distinct absorption peak emerged at 942 cm^{-1},[8] corresponding to the B–O stretching vibrations characteristic of the boronate ester linkage. This peak is indicative of the covalent interaction between the –OH groups of CB and the *cis*-diol groups of SA, affirming the successful formation of the boronate ester linkage under the tested conditions. These findings collectively validate the efficient conjugation of CB with SA through the formation of stable boronate ester linkages within a tumor-mimicking microenvironment, thereby highlighting the hydrogel's suitability for targeted therapeutic applications.

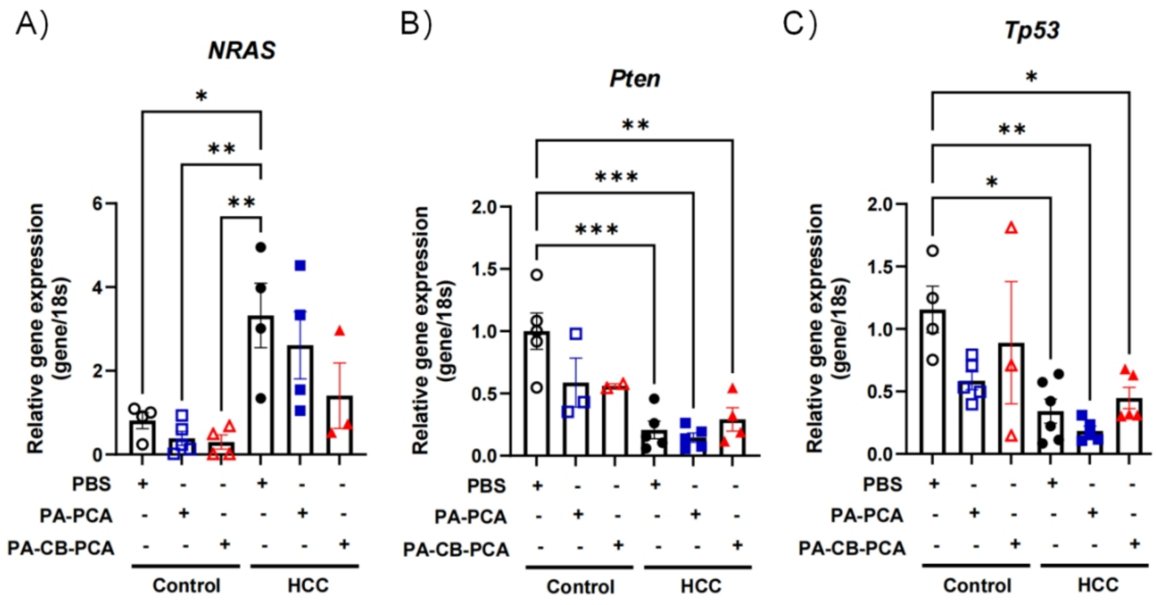

Figure S9. qRT-PCR validation of gene expression changes in the hepatocellular carcinoma (HCC) mouse model following plasmid injection and treatments. (**A**) Relative expression of the oncogene *NRAS* in liver tissues from PBS-treated HCC, PA–PCA-treated HCC, PA–CB–PCA-treated HCC, healthy control, and untreated HCC groups. (**B**) Relative expression of the tumor suppressor gene *Pten*. (**C**) Relative expression of the tumor suppressor gene *Tp53*.

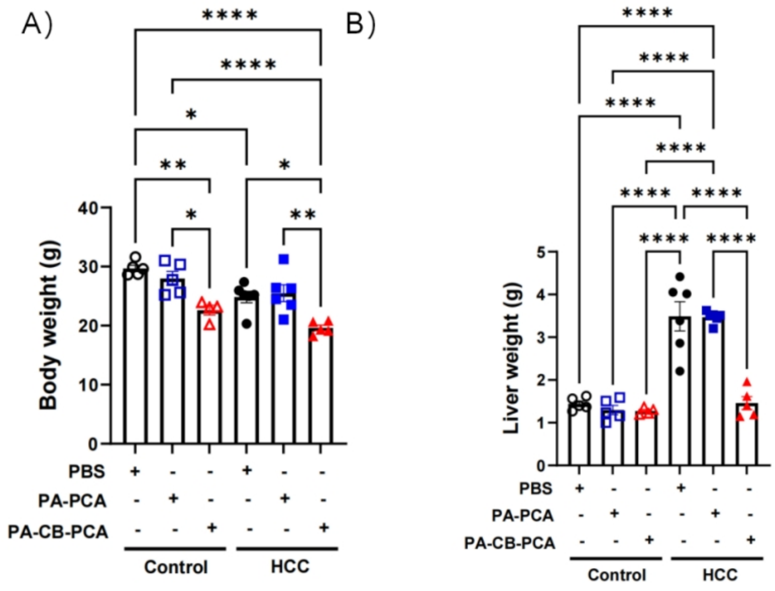

Figure S10. Body and liver weights in the HCC mouse model following treatments. (**A**) Body weights of mice from PBS-treated HCC, PA–PCA-treated HCC, PA–CB–PCA-treated HCC, healthy control, and untreated HCC groups. (**B**) Liver weights in the same groups.

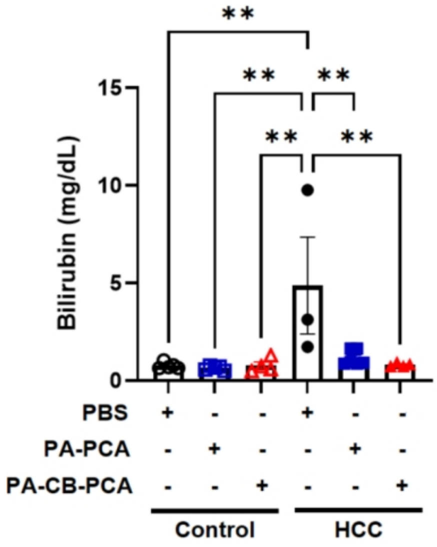

Figure S11. Serum total bilirubin levels in the HCC mouse model following treatments.

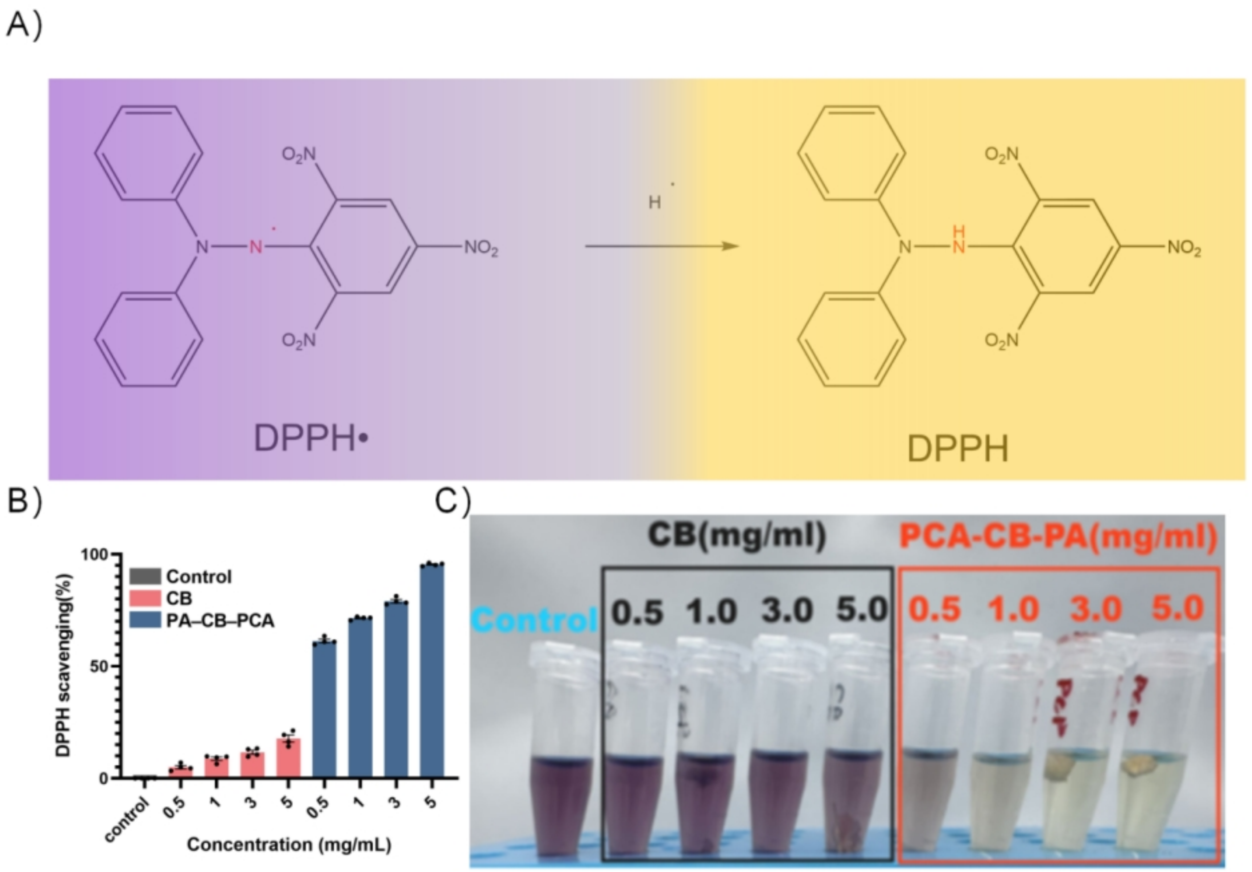

Figure S12. *In vitro* antioxidant activity of PA–CB–PCA evaluated using the DPPH radical scavenging assay. (A) Schematic representation of the reduction of DPPH radicals (DPPH•) to the nonradical form (DPPH) through hydrogen atom donation. (B) Dose-dependent DPPH scavenging percentage for the control, CB, and PA–CB–PCA groups at concentrations ranging from 0.1 to 0.7 $mg\cdot mL^{-1}$. (C) Photographic images illustrating the color change (from purple to yellow) in DPPH solutions upon treatment with increasing concentrations (0.5–5.0 $mg\cdot mL^{-1}$) of CB and PA–CB–PCA, relative to the control. Data are presented as the means ± SD (n = 4 for (B)).

REFERENCES

[1] X. Wang, H. Tang, C. Wang, J. Zhang, W. Wu, X. Jiang, Phenylboronic acid-mediated tumor targeting of chitosan nanoparticles, Theranostics 6 (2016) 1378 – 1392. https://doi.org/10.7150/thno.15156.

[2] J. Li, W. Hu, Y. Zhang, H. Tan, X. Yan, L. Zhao, H. Liang, pH- and glucose-dually responsive injectable hydrogel prepared by in situ crosslinking of phenylboronic acid-modified chitosan and oxidized dextran, J. Polym. Sci. A Polym. Chem. 53 (2015) 1235 – 1244. https://doi.org/10.1002/pola.27556.

[3] H. Du, X. Liu, X. Sun, H. An, Y. Li, Z. Zhu, Y. Wen, CO_2 -responsive multifunctional label based on chitosan and hyaluronic acid for visualizing and maintaining postharvest freshness, Food Hydrocoll. 157 (2024) 110438. https://doi.org/10.1016/j.foodhyd.2024.110438.

[4] A. Li, W. Chen, H. Shi, Y. Ye, P. Gong, B. Jiang, B. Xiao, Synthesis, properties and applications of a polyampholyte hydroxypropyl chitosan derivative with the phenylboronic acid functional group, Int. J. Biol. Macromol. 258 (2024) 128721. https://doi.org/10.1016/j.ijbiomac.2023.128721.

[5] Y. Yu, L. Zhang, B. Hu, Z. Wang, Q. Gu, W. Wang, C. Zhu, S. Wang, Borate bonds-containing pH-responsive chitosan hydrogel for postoperative tumor recurrence and wound infection prevention, Carbohydr. Polym. 339 (2024) 122262. https://doi.org/10.1016/j.carbpol.2024.122262.

[6] T. Sainsbury, A. Satti, P. May, Z. Wang, I. McGovern, Y.K. Gun’ko, J. Coleman, Oxygen radical functionalization of boron nitride nanosheets, J. Am. Chem. Soc. 134 (2012) 18758 – 18771. https://doi.org/10.1021/ja3080665.

[7] J.-Z. Wu, G.R. Williams, H.-Y. Li, D. Wang, H. Wu, S.-D. Li, L.-M. Zhu, Glucose- and temperature-sensitive nanoparticles for insulin delivery, Int. J. Nanomedicine 12 (2017) 4037–4057. https://doi.org/10.2147/IJN.S132984.

[8] M.K. Smith, B.H. Northrop, Vibrational properties of boroxine anhydride and boronate ester materials: Model systems for the diagnostic characterization of covalent organic frameworks, Chem. Mater. 26 (2014) 3781–3795. https://doi.org/10.1021/cm5013679.

[9] Z. Chen, W. Zhang, C.-A. Palma, A. Lodi Rizzini, B. Liu, A. Abbas, N. Richter, L. Martini, X.-Y. Wang, N. Cavani, H. Lu, N. Mishra, C. Coletti, R. Berger, F. Klappenberger, M. Kläui, A. Candini, M. Affronte, C. Zhou, V. De Renzi, U. del Pennino, J.V. Barth, H.J. Räder, A. Narita, X. Feng, K. Müllen, Synthesis of graphene nanoribbons by ambient-pressure chemical vapor deposition and device integration, J. Am. Chem. Soc. 138 (2016) 15488–15496. https://doi.org/10.1021/jacs.6b10374.

[10] M. Serafini, C. Cordero-Sanchez, R. Di Paola, I.P. Bhela, S. Aprile, B. Purghè, R. Fusco, S. Cuzzocrea, A.A. Genazzani, B. Riva, T. Pirali, Store-operated calcium entry as a therapeutic target in acute pancreatitis: Discovery and development of drug-like SOCE inhibitors, J. Med. Chem. 63 (2020) 14761–14779. https://doi.org/10.1021/acs.jmedchem.0c01305.

[11] S. Aras, F.C. Cavusoglu, D. Unlu, Synthesis, characterization and thermal pyrolysis mechanism of resorcinol–formaldehyde resin-containing phenylboronic acid, Adv. Eng. Mater. 26 (2024) 2400285. https://doi.org/10.1002/adem.202400285.

[12] M. Dash, F. Chiellini, R.M. Ottenbrite, E. Chiellini, Chitosan—a versatile semi-synthetic polymer in biomedical applications, Prog. Polym. Sci. 36 (2011) 981–1014. https://doi.org/10.1016/j.progpolymsci.2011.02.001.

[13] S. Lu, X. Song, D. Cao, Y. Chen, K. Yao, Preparation of water-soluble chitosan, J. Appl. Polym. Sci. 91 (2004) 3497 – 3503. https://doi.org/10.1002/app.13537.

[14] C. Qin, H. Li, Q. Xiao, Y. Liu, J. Zhu, Y. Du, Water-solubility of chitosan and its antimicrobial activity, Carbohydr. Polym. 63 (2006) 367 – 374. https://doi.org/10.1016/j.carbpol.2005.09.023.

[15] C. Yan, R. Liu, C. Zhang, Y. Cao, X. Long, Synthesis and formation mechanism of submicrometer ZrB_2 powders via the Pechini-type polymerizable complex route, RSC Adv. 5 (2015) 78606 – 78613. https://doi.org/10.1039/C5RA13010G.

[16] Y. Ding, D. Liu, Y. Sun, S. Liu, P. Wang, S. Wang, D. Huang, J. Ji, Dynamic imine bond-enabled starch-based materials with self-healing and recycling properties, ACS Sustain. Chem. Eng. 13 (2025) 6388 – 6398. https://doi.org/10.1021/acssuschemeng.5c01440.

[17] Z. Su, L. Cui, H. Zhang, L. Xiao, B. Chi, H. Xu, L. Ning, S. Jia, X. Wang, Robust, waterproof and degradable cellulose-based polyimine vitrimer for plastic replacement, Chem. Eng. J. 471 (2023) 144501. https://doi.org/10.1016/j.cej.2023.144501.

[18] Y. Oyarzún, J. Ulloa, M. Ceballos, B.F. Urbano, Dynamic covalent boronic-acid-functionalized alginate/PVA hydrogels for pH- and shear-responsive drug delivery, Gels 10 (2024) 504. https://doi.org/10.3390/gels10080504.

[19] J.L.G. López, P. Schmieder, K. Kemnitz-Hassanin, H.C. Asikoglu, A. Celik, C.E. Stieger, D. Fiedler, S. Hinderlich, C.P. Hackenberger, Real-time monitoring of the sialic acid biosynthesis pathway by NMR, Chem. Sci. 14 (2023) 3482 – 3492. https://doi.org/10.1039/D2SC06986E.

[20] H. Otsuka, E. Uchimura, H. Koshino, T. Okano, K. Kataoka, Anomalous binding profile of phenylboronic acid with N-acetylneuraminic acid (Neu5Ac) in aqueous solution with

varying pH, J. Am. Chem. Soc. 125 (2003) 3493 – 3502. https://doi.org/10.1021/ja021303r.

[21] J.-J. Max, C. Chapados, Infrared spectroscopy of aqueous carboxylic acids: Malic acid, J. Phys. Chem. A 106 (2002) 6452 – 6461. https://doi.org/10.1021/jp014377i
